# A Spatially Continuous Model of Carbohydrate Digestion and Transport Processes in the Colon

**DOI:** 10.1371/journal.pone.0145309

**Published:** 2015-12-17

**Authors:** Arun S. Moorthy, Stephen P. J. Brooks, Martin Kalmokoff, Hermann J. Eberl

**Affiliations:** 1 Biophysics Interdepartmental Group and Department of Mathematics & Statistics, University of Guelph, Guelph, Ontario, Canada; 2 Bureau of Nutrition, Food Directorate, Health Canada, Tunney’s Pasture, Ottawa, Ontario, Canada; 3 Atlantic Food and Horticulture Research Station, Agriculture and Agri-Food Canada, Kentville, Nova Scotia, Canada; Argonne National Lab, UNITED STATES

## Abstract

A spatially continuous mathematical model of transport processes, anaerobic digestion and microbial complexity as would be expected in the human colon is presented. The model is a system of first-order partial differential equations with context determined number of dependent variables, and stiff, non-linear source terms. Numerical simulation of the model is used to elucidate information about the colon-microbiota complex. It is found that the composition of materials on outflow of the model does not well-describe the composition of material in other model locations, and inferences using outflow data varies according to model reactor representation. Additionally, increased microbial complexity allows the total microbial community to withstand major system perturbations in diet and community structure. However, distribution of strains and functional groups within the microbial community can be modified depending on perturbation length and microbial kinetic parameters. Preliminary model extensions and potential investigative opportunities using the computational model are discussed.

## Introduction

The gastro-intestinal tract (GIT) describes the collection of organs involved in converting ingested materials into usable energy sources while removing additional waste products. Digestion predominantly occurs in the upper GIT (mouth, stomach, small intestine, etc) while final nutrient extraction and fecal preparation (water absorption) occurs in the lower GIT [1]. Energy extraction from dietary materials is of importance to the proper function of all other physiological systems. The large intestine, or colon, is the primary organ of lower GIT. It can be described as a large, muscular passage (lumen), lined with irregular villi structures covered in endogenously produced mucous fluid, and is host to upwards of 400 species of microbes [2]. The anaerobic environment supports intestinal microbiota growth, providing the microbes with fermentable substrates, and in return, the microbial community increases host biochemical capabilities, mediates gut epithelial cell differentiation, and influences host physiological development [3]. Over the past several years, suggestions surrounding the intestinal microbiota and its role in many aspects of health have been prevalent—from general physiology and development [4–7], to more particular concerns such as brain health [8] and cancer [9].

The microbiota residing in the human colon is inherently difficult to investigate due to its physical inaccessibility; often assessment is done using only the materials entering the digestive system through diet [10] or exiting as feces [11]. As such, many laboratory models, both *in vivo* and *in vitro*, have been developed and used extensively [12, 13] for experimental investigation. Most reactor representations work under the assumption that the colon can be well approximated using a sequence of chemical reactors connected serially [14–16]. The number of reactors and levels of physiological mimicry (e.g. mucus representation, microbiota density) varies greatly between experiments as discussed in [13] and references therein.

In [12], the authors note that the robustness of the human colonic flora allows it to be studied in great depth and with significant relevance using *in vitro* models, however, suggest the following limitations: (i) Models do not incorporate host immune or neuroendocrine system functionality. (ii) Other biotic factors are not incorporated into the models (e.g. gut absorptive processes, digestive tract secretions or defensive systems). (iii) Epithelial and other colonic biofilms are not reproducible in the models. (iv) It is difficult to control changes in microbiota community structure in the models following inoculation, particularly in closed systems.

An additional approach to investigating biological systems used in combination with *in vitro* and *in vivo* modeling is the development of mathematical models. Mathematical models currently used in investigating properties of colon function provide a similar level of abstraction as *in vitro* models, greatly simplifying the geometric/physical representation of the colon and often focusing on the microbiology of the anaerobic digestion processes that occur [17, 18], or as proof-of-concept tools for specific health scenarios [19, 20]. This classical approach to building mathematical models of biological systems allows for the creation of small models, suitable for rigorous analysis and/or verification using controlled experimental protocols.

To the best of our knowledge, the mathematical model of carbohydrate degradation presented in [18], herein referred to as the MT model, is the most complete model of anaerobic digestion in physical environments mimicking the colon. The MT model builds upon the Anaerobic Digestion Model No. 1 (ADM1) [21] framework for describing biochemical and physico-chemical processes involved in anaerobic digestion. The MT model assumes carbohydrates to be the sole substrate entering the colon (disregarding proteins, lipids, and subsequent metabolites), and that distinguishing microbes by their functional involvement as biomass functional groups (BFGs) rather than traditional biological taxa is sufficient in capturing the effective capabilities of the microbial communities, resulting in the digestion processes being described in 17-state variables (sugar, lactate, acetate, propionate, butyrate, dissolved hydrogen, hydrogen gas, dissolved methane, methane gas, dissolved carbon dioxide, carbon dioxide gas, polysaccharide, sugar utilizing biomass, lactate utilizing biomass, acetogens, methanogens)[18]. Additionally, the MT model attempts to capture the physical structure of the colon in a manner similar to a 3-stage reactor system with porous polysaccharide matrix to immobilize microbial cultures, distinguishing three physical locations (proximal, transverse, distal colon) and two biochemical environments (lumen and mucus)—leading to a final system of 102-ODEs describing anaerobic digestion in the colon.

Following our work assessing the effect of physical reactor representations in the MT model, in which we used computational representations of the MT model implemented as a single stage reactor, 3-stage reactor system, and 3-stage reactor system with volume controlled outflow, we concluded that there was limited effect of volume controlled outflow on the microbiota composition between the compared systems [22]. However, the microbiota composition was sensitive to number of stages, motivating our investigation of a plug-flow reactor representation. It was also noted in Williams et. al [23] that a limitation of current *in silico* models is the lack of plug flow representation. Subsequently, we have developed a spatially continuous framework for modeling processes in the human large intestine; analogous to a continuous pipe plug flow reactor (PFR) with a fluid medium transporting through the pipe, and a fixed-medium of constant volume attached to pipes inner surfaces.

Many of the more than 400 different species of microbes are functionally redundant (reaction activity), yet the abundance and composition of this intestinal community has been suggested as an important contributor to health; see [4] and references therein. In [24], the authors extend the ADM1 model framework to simulate *species* of microbes within a biomass functional group. In particular, each biomass functional group within the ADM1 model was subdivided into 10 representatives where species (or strains) can be identified within a group based on their specified biochemical reaction parameters. We adapt a similar idea to extend the MT model of digestion in the colon to consider multiple strains as well. By adding microbial complexity, we allow ourselves to explore a wider set of hypotheses that are pertinent to biological literature, such as the effects of perturbations within biomass functional groups, than the MT model is capable at present. Herein, we refer to this extended model as the *e*MT model.

Combining the previously described PFR-type reactor representation and the *e*MT model of carbohydrate digestion, we have created a spatially continuous mathematical model of transport processes, anaerobic digestion, and microbial complexity as would be expected in the large intestine. The resulting model is a system of first-order PDEs with context dependent number of state variables and stiff, non-linear source terms. is not amendable to rigorous qualitative mathematical analysis, but a software implementation, compuGUT (http://compugut.sourceforge.net), is made available for quantitative computer simulation and qualitative validation (trends versus measurements). The software tool is flexible and extensible, providing a framework from which we can uncover insights that guide further detailed/refined mathematical modeling, and pose questions that may encourage further experimental development and/or investigation.

In this paper, we use our computational model to demonstrate (i) the importance of reactor representation in describing colon material composition, particularly when attempting to infer information about proximal colon locations using outflow data, (ii) the ability of complex (rich) microbial communities to buffer the impact of input (dietary) perturbations, and (iii) the ability of complex microbial communities to withstand extreme environmental/physiological changes. Finally, we discuss the general strengths and limitations of our model, proposing opportunities, extensions and potential experimental inquiry.

## Materials and Methods

The large intestine is the distal most portion of the gastro-intestinal tract. It is responsible for the final absorption of digestive nutrients and preparation of fecal matter for bowel movements [1]. It is an open tube-like organ with muscular walls to aid in the continued transport of eventual waste materials. The walls of the colon are also lubricated with endogenously produced mucus. The colon is often described in three separate locations: the proximal (or ascending), transverse, and distal (or descending) colon. These three locations have differing physical conditions, specifically with regard to the acidity (with locations closer to the proximal end being more acidic than towards the distal end) and the absorption/transportation rates at which substrates are removed from the colon [1]. The colon’s biochemical environment makes it a highly suitable habitat to dense communities of microbiota. One of the primary functions of the intestinal microbiota is to digest chemically indigestible materials (such as dietary fiber). Metabolites generated through this digestion process are absorbed by the gut, and waste material is transported along the length of the colon. Thus, we can think of colon functionality as being defined by three sub-processes with dynamics governed by the interaction of a complex network of microbiota, substrates, metabolites and physical forces, in multiple physically and biochemically diverse environments: (i) the digestion of particulate material, (ii) the exchange of soluble materials between biochemical environments (lumen-mucus-host), and (iii) the convective transport of materials through the length of the colon.

**Fig 1:**
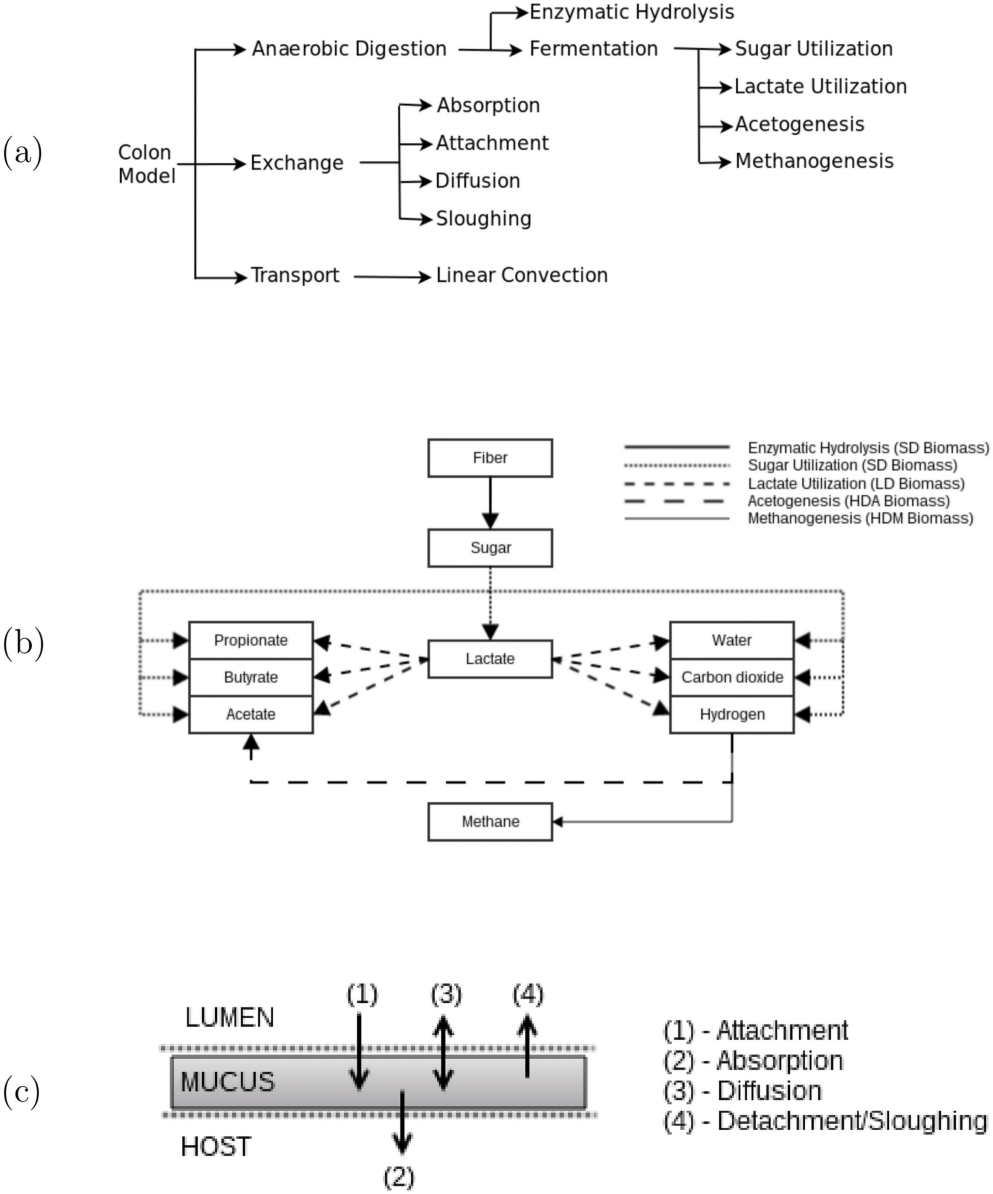
Overview of compuGUTs underlying mathematical models. (a) Schematic overview of biochemical and physical sub-processes considered in the compuGUT. (b) 5-step model of anaerobic digestion, adapted from [18]. Biomass functional group active in each step indicated in parentheses. (c) Summary of component exchange processes. Material in the lumen environment is transported along the length of the colon where as mucus material is stationary along the length of the colon and only experiences axial transport.

By way of material balance, we can combine the three sub-processes and describe the density of materials in the colon with the following advection-reaction system:

$$\begin{array}{c} \partial_{t}c+\partial_{x}F\left(c\right)=R\left(c\right)+E\left(c\right) \end{array}$$ (1)

where the functions *R*, *F* and *E* can be interpreted as non-linear functions describing the sub-processes of anaerobic digestion, material transport, and component exchange, respectively, and their input, **c**, is a vector of concentrations [g/L] of all materials considered in the colon-complex. We describe functions *R*, *F* and *E* in detail in the following sections, but present Fig 1a as a schematic representation of the model structure and foundational processes.

### Assumptions

Physiological systems are highly complex, functioning with redundancy, time-variations and interplay with other systems [25]. Rather than model the entire physiology of the colon, we look to capture the integral mechanisms defining the colon-diet-flora system with as little complexity as possible. We introduce the following simplifying assumptions for model development:

- **Colon Geometry**: A cross-sectional slice of the colon would display highly irregular geometry, as there exists mucousal folds and villous surfaces [26, 27]. For simplicity, the geometry of the large intestine is averaged as a cylindrical tube of constant diameter. Combining this simplification with the knowledge that the length of the colon is significantly larger than its diameter allows us to model the colon as 1-dimensional in space (x-dimension)
- **Material Properties**: To be consistent with our first assumption (1D tube geometry), we assume that the materials in localized portions of the colon (a particular x-value) will be homogeneous (well-mixed) across the cross-sectional area. Additionally, we only measure water generated through the fermentation process, rather than try to account for all water balances through interaction with other physiological processes.
- **Mucus Thickness**: Mucus is produced endogenously throughout the colon. The rate of this mucus production is constant along the main flow direction and across the mucus thickness. Additionally, we treat this layer as a fixed medium of constant volume, with the volume of the mucus being 10% of the total colon volume.
- **Transit Time**: The effect of peristalsis and additional propulsion mechanisms manifest as an average flow or speed of convective transport. This allows us to approximate convective transport as a first-order flux with constant velocity term.
- **Metabolic Pathways**: The only macromolecules reaching the colon are carbohydrates, and the anaerobic digestion of carbohydrates follows the metabolic pathway described in [18]. As a strict carbohydrate digestion model, other materials that compose mucus (proteins, lipids) are not considered in calculations. This metabolic pathway can be summarized as a five-step process (highlighted in Fig 1b), where fiber is first hydrolyzed to monomer sugars, and then monomer sugars are fermented by intestinal microbiota into various metabolites (lactate, acetate, propionate, butyrate, hydrogen, methane, carbon dioxide, and water) in the parallel processes of sugar utilization, lactate utilization, acetogenesis and methanogenesis. Although there are over 400 species of microbes inhabiting the colon [2, 3], we assume that the total colon bacterial flora can be sub-divided into four biomass functional groups according to fermentative pathway. We define flora as either Sugar Fermenters (SD), Lactate Fermenters (LD), Hydrogen Oxidizing Acetogens (HDA), or Hydrogen Oxidizing Methanogens (HDM). Hydrolysis progresses due to enzymes produced by SD flora. Assuming micobial group functionality as a sufficient means to categorize microbiota is a familiar approach in microbial modeling and waste water engineering [18, 21, 28].
- **Reaction Processes**: Combining the processes involved in metabolism and the natural decay of flora in the system, we can summarize the reaction processes in the flora-diet system as: (1) hydrolysis, (2) glucose utilization, (3) lactate utilization, (4) acetogenesis, (5) methanogenesis, (6) decay of SD flora, (7) decay of LD flora, (8) decay of HDA flora, and (9) decay of HDM flora. The choice of metabolic pathways and subsequent reaction processes is responsible for the overall model problem size, thus a simpler representation of anaerobic digestion would lead to a smaller state-space, and a more involved representation of anaerobic digestion would lead to a larger state space.

We remark that the model assumptions and simplifications can be relaxed on future model iterations as knowledge of functional details continues to grow, but doing so would require the inclusion of additional mathematical and numerical complexities. Complete mathematical formulation and details are provided in S1 Appendix.

The choice of sub-models, particularly of anaerobic digestion/metabolic pathway, is key to determining the size and structure of the overall model. We build upon the model of [18] which describes the degradation of carbohydrates in the context of the human colon. A schematic representation of the modeled anaerobic digestion process is shown in Fig 1b, where fibers are converted to sugars and subsequent metabolites in the presence of functional biomass groups. The sub-model of component exchange processes is presented as Fig 1c, where solutes (SCFA, simple compounds) undergoes attachment and absorption, biomass undergo attachment and detachment, mucus fibers undergo sloughing, and sugars diffuse between lumen and mucus environments. Materials in the lumen are transported along the colon, where as mucus material is fixed to the colon wall and so the sub-model of transport is a defined using linear convection with an average flow rate value representing convective velocity for lumen components and a convective velocity of zero for mucus components. It should be noted that processes in GIT upstream of the colon are not explicitly modeled. Instead, a *blackbox* approach to describe the upper GIT as a system of dilution-units is employed. This approach effectively assumes there is continuous flow through and limited biochemical changes to material in the upper GIT.

### compuGUT

The developed mathematical model of context driven problem-size and functionally defined sub-processes presents significant organizational challenges during numerical simulation. Additionally, simulation of large models will invariably create large data-sets, both with analytical and visualization challenges. The compuGUT software project stems from these design challenges, providing interested users a preliminary model implementation for review and experimentation. The compuGUT suite of tools was developed for Linux systems, and performs three primary tasks: (1) Simulation Design, (2) Numerical Integration, and (3) Data Visualization. Tasks (1) and (3) are performed by auxiliary tools written as either BASH or R scripts, while task (2) is performed by our primary compuGUT tool written in C, which is a flexible implementation of a standard central-flux scheme for balance laws [29]. The compuGUT requires traditional c-libraries (stdlib, stdio, math, string, time, errno) as well as external libraries, namely, the sundials package for solving non-linear problems [30]. The compuGUT interactive visualization tools make use of the googleVis API [31]. As of now, the compuGUT has been tested on 32 and 64-bit Linux workstations. Source codes, user-friendly operation and visualization scripts, additional files and resources, as well as pre-compiled 32 and 64 bit Linux binaries are available under GNU GPLv3 licensing. Technical details regarding the software implementation, including numerical methods and validation, are provided as technical documentation on the project home page (http://compugut.sourceforge.net).

### Simulation Scenarios and Analytical Techniques

To study the features of the spatial continuity and *e*MT carbohydrate digestion model, we execute a series of simulation experiments/scenarios using the compuGUT. Source codes and analytical scripts used to perform and study the following simulation scenarios are available on the compuGUT project page.

#### Spatial Reactor Representation

As noted, the model of [18] assumes the colon can be represented by a system of three sequential reactors. We use the compuGUT simulation tool to simulate both a three-stage reactor representation and spatially continuous reactor under various system flow rate and dietary fiber conditions. To ensure both models are comparable, we assess spatial representation while using the MT model to describe carbohydrate digestion (single representation within a biomass functional group). We look at five mean-transit times, the amount of time required for material to travel the complete length of the colon: (a) 6 hours, (b) 12 hours, (c) 24 hours, (d) 48 hours, and (e) 96 hours. The average transit-time for a healthy colon is between 12 and 48 hours. We include assessment of 6 and 96 hours to simulate extreme/unhealthy systems (analogous to expected flow conditions during diarrhea or constipation). For each transit time, we consider three dietary fiber intake volumes: (a) 15, (b) 30, and (c) 45 grams per day, input as three meals of length 15 minutes. The recommended daily fiber intake is 25 grams per day. The result is a total of 15 simulations (5 flow values x 3 fiber intakes). Each scenario is simulated for 56 days.

To analyze the performance of both reactor representations, we extrapolate the solution of the three-stage reactor over the length of the entire colon, and then compare the difference in their prediction of material composition, particularly percentage of total material that is a biomass quantity, as a percent deviation:

$$\text{\%Deviation = }\frac{{\text{\%Biomass}}_{\text{D}}-{\text{\%Biomass}}_{\text{C}}}{{\text{\%Biomass}}_{\text{D}}}\text{,}$$ (2)

where %Biomass_D_ and %Biomass_C_ are the percent biomass values generated at all locations and time steps using the 3-stage discrete physical model and the spatially continuous physical model, respectively, and percent biomass is tabulated as:

$$\begin{array}{c} \text{\%Biomass}=\frac{\text{Biomass}}{\text{Total}\;\text{Mass}}, \end{array}$$ (3)

where biomass is the sum of sugar utilizing, lactate utilizing, acetogens and methanogens in both the lumen and mucus environments, weighted for volume differences, and total mass includes biomass and all other components (fiber, sugars, short-chain fatty acids, and simple compounds).

#### Microbial Refinement

By extending the description of carbohydrate digestion of [18] to include more representation within biomass functional groups, we introduce additional complexity.

To assess the value of added microbial complexity beyond parameter sensitivity, we simulate hypothetical system scenarios with differing microbial representations. Particularly, we look at how the entire colon-complex responds to a sudden/temporary drop off in dietary fiber consumption, or to a sudden/temporary removal of the major or predominant strains/representatives within the sugar utilizing biomass functional group.

**Fig 2:**
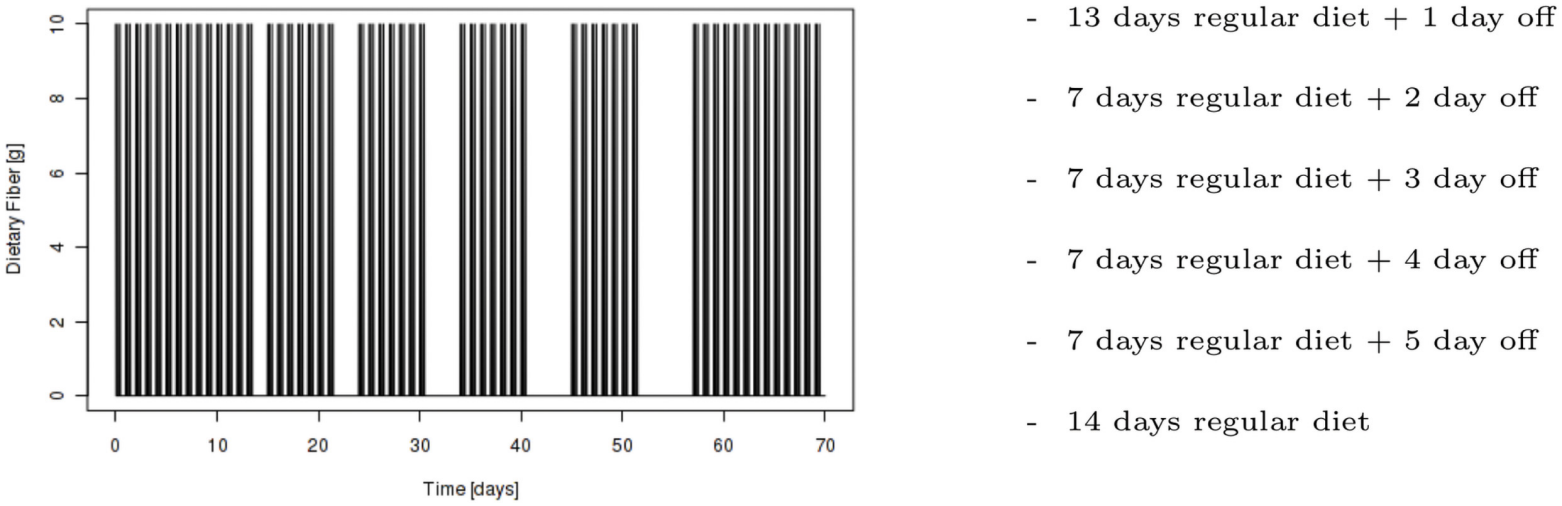
Dynamic profile of dietary fiber consumption in diet perturbation simulation experiments.

For diet perturbations, we execute a 10 week simulation with diet profile as outlined in Fig 2, where a regular diet is defined as consuming 30 grams of fiber per day split over 3 equal meals at hours 0, 4, and 10 (i.e. 8am, 12pm, 6pm), and off diets indicate no dietary fiber consumption. This simulation is undertaken with 4 levels of microbial refinement, namely: 1, 2, 5, and 10 representatives within the sugar utilizing biomass functional group. Simulation scenarios were run with a biochemical deviation of 10% and are repeated 10 times to average the effects of the stochastically chosen parameters.

To analyze diet perturbations, we compare the average percent biomass (as calculated previously) produced in each of the 4 microbiota representations (1, 2, 5 and 10 representations in the sugar utilizing biomass functional group) for all locations along the length of the colon and at all times during the entire simulation.

Microbial communities can be disturbed due to a variety of physiological and/or environmental conditions. We define microbial community perturbation in our simulations as an an increased cell lysis rate of the dominant representative strain in the sugar utilizing biomass functional group. An alternative approach to community perturbation could be increased removal rates (washout) of a dominant strain, but given the current stage of our flow model development, increasing cell lysis rate was considered a convenient means to perturb the microbial system in a easily quantifiable way. We simulate systems of 5 sugar degraders, and perturb each system for a fixed number of days (between 0 and 10). To analyze the output of these simulations, we first look at the percent biomass (as described previously) at the output of the colon to assess the macroscopic behavior of the system, and then look at the detailed community structure by assessing the percent composition of each of the 5 sugar utilizing biomass representatives. Simulation scenarios were run with a biochemical deviation of 1% and are repeated 6 times to evaluate the effects of stochastically chosen parameters.

## Results

**Fig 3:**
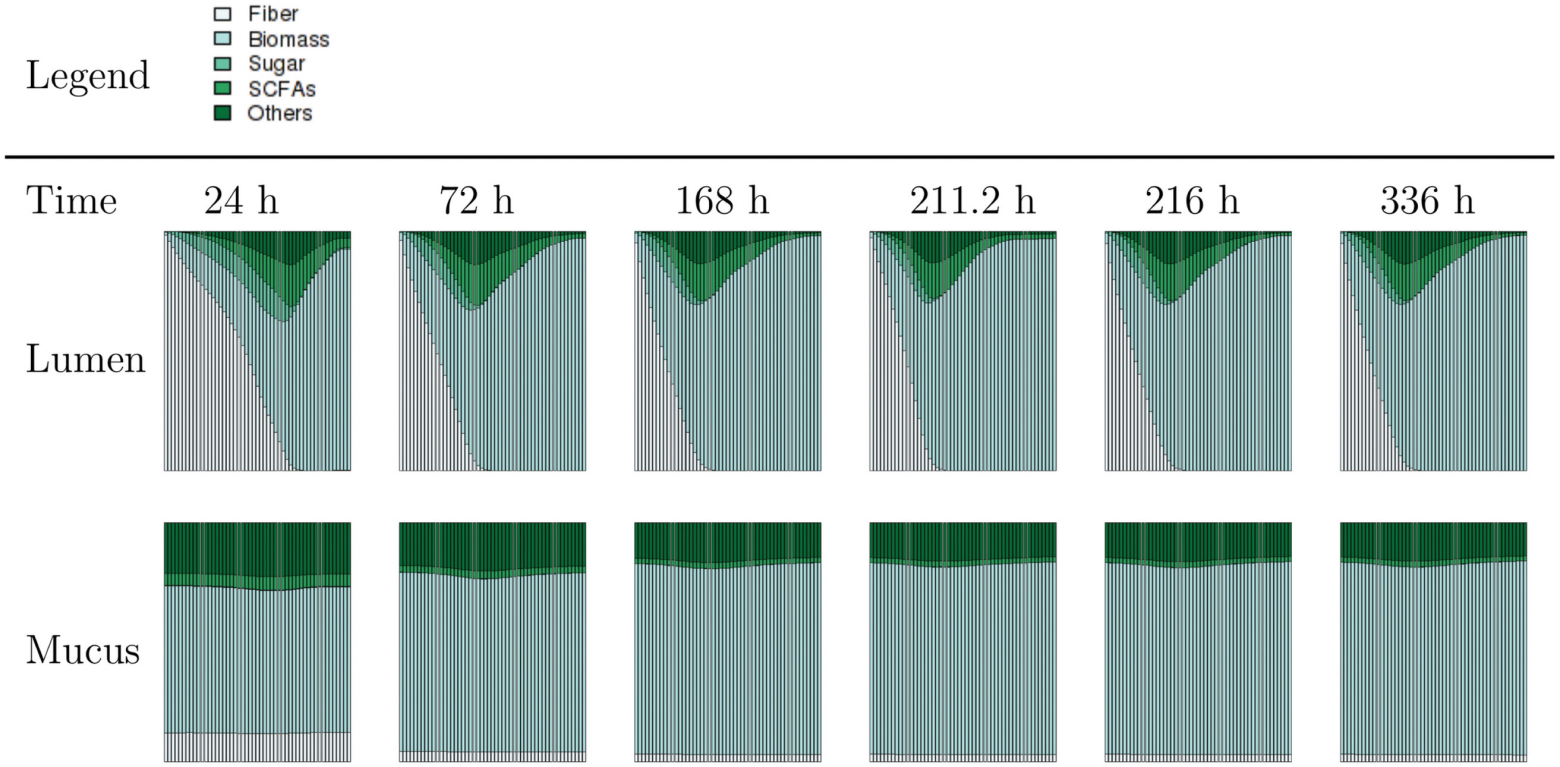
Example of summary plots generated using a basic simulation of the compuGUT. Plots illustrate the coarse composition of material (as percentage) along the length of the colon (x-axis), at various time values during an 8 week simulation. Complete time dynamics animations can be found on project web-page. Figures in rows 1 and 2 indicate solution at specified times in the lumen and mucus environments, respectively. These figures demonstrate the relative consistency of material composition along the length of the colon in the mucus environment, and that material composition changes notably along the length of the colon in the lumen environment. However, the profile along the length of the colon in the lumen environment does approach a periodic solution (best illustrated as animation) after approx. 2 weeks (336 hours).

A single 8 week simulation of the compuGUT with single representatives within biomass functional groups (MT model), normal physiological conditions (volume, flow rate), and coarse-most numerical discretization generates 2821 text files with information regarding the concentration of all state-variables at all locations. It should be noted that the anaerobic digestion model used for simulation only includes gas components in dissolved form rather than both dissolved and vapor. This results in concentrations of 28 dependent variables at the minimum 51 colon locations. A visualization of coarse-composition, which we define as material categorized by type as either fiber, biomass, sugar, short-chain fatty acids (SFCAs) or others, is shown as Fig 3 as demonstration of the types of visual tools used to explain, describe, and reason simulation experiment results; we refer to these visualizations as exploratory plots/animation. Following, we present a description of simulation experiment results in a comprehensive manner, however, the exploratory animations used to contrive these descriptions can be found on the project web-page.

### Effect of reactor representation

**Fig 4:**
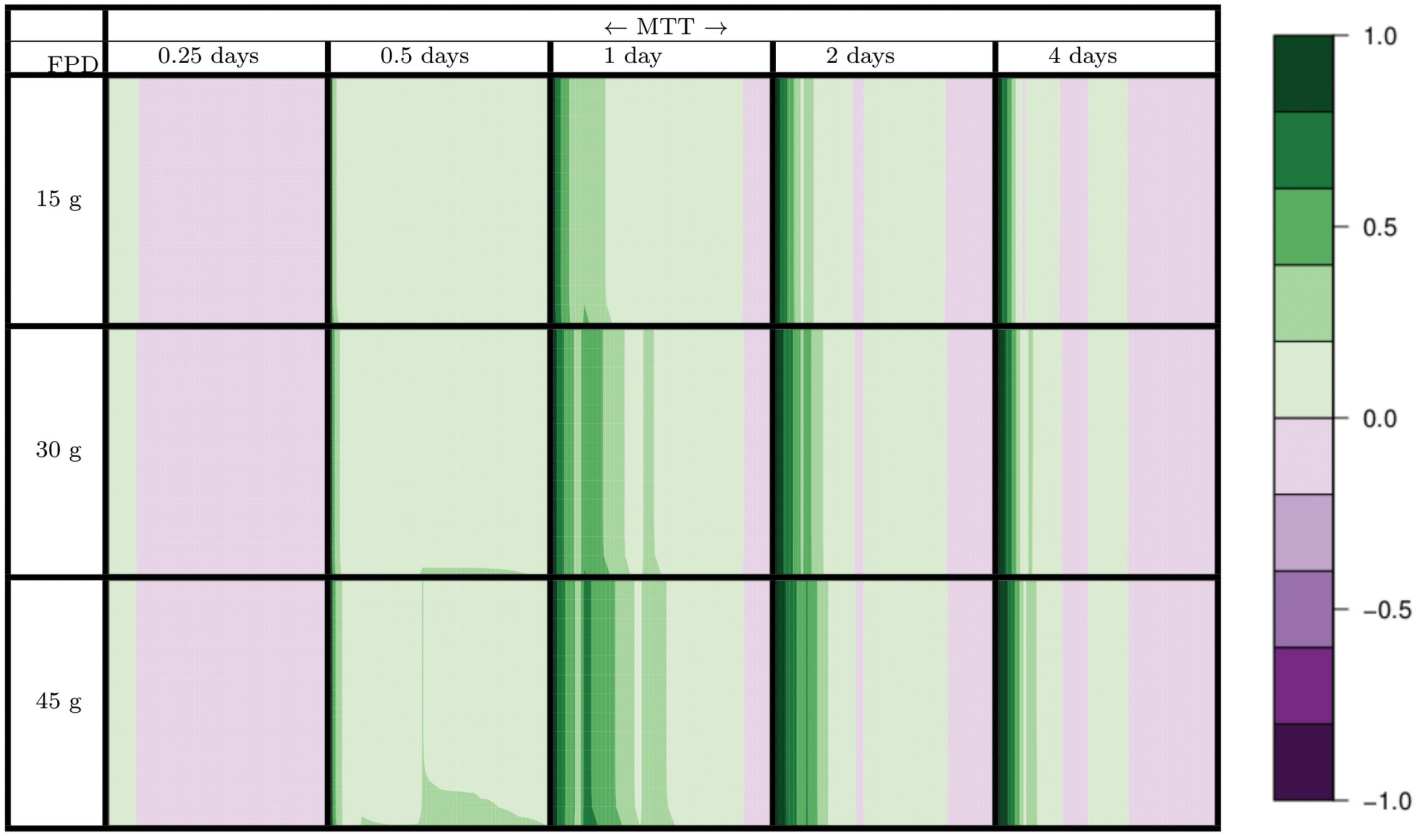
Matrix of plots showing deviation between 3-stage reactor system and spatially continuous model in measuring coarse material composition (particular percent biomass) for 15 fiber/flow scenarios (as described in the Materials and Methods) categorized by 5 mean transit times (MTT) coupled with 3 daily fiber intakes (FPD). Each plot within matrix shows deviation along the length of the colon (x-axis) through an 8 week simulation (y-axis). Figure legend applies to each plot, where green indicates the 3-stage reactor over predicts the continuous model, and purple indicates the 3-stage reactor under predicts the continuous model.

In assessing the coarse composition of material along the length of the colon using both the three-stage physical representation and the spatially continuous representation, we find substantial differences between both model outputs. Fig 4 illustrates the deviation Eq (2) between the three stage model and the continuous model, where green and purple indicate over and under-prediction, respectively, with a gradient between differences. In this case, we look at the percent biomass in the total material composition across the length of the colon (x-axis) over the entire simulation time (y-axis) for 15 flow/fiber scenarios described in the Materials and Methods. We display the results as an array of plots, where scenarios are organized by Mean Transit Time (MTT) in columns, and Fiber Intake per Day (FPD) as rows. In scenarios with low transit times (fast flow rate), the largest inter-model difference is at the distal end of the reactor, where the three-stage representation over predicts the coarse material composition measured in the continuous representation. In scenarios with average to high transit times (standard to slow flow rates), largest inter-model difference is at the proximal end of the reactor, where the three-stage representation over predicts the coarse material composition measured in the continuous representation. Also, inter-model difference is magnified in simulations with larger fiber intakes. This is due to increased substrate availability leading to increased reaction progress, furthering the spread between models.

### Effect of microbiota refinement

#### Diet Perturbation

**Fig 5:**
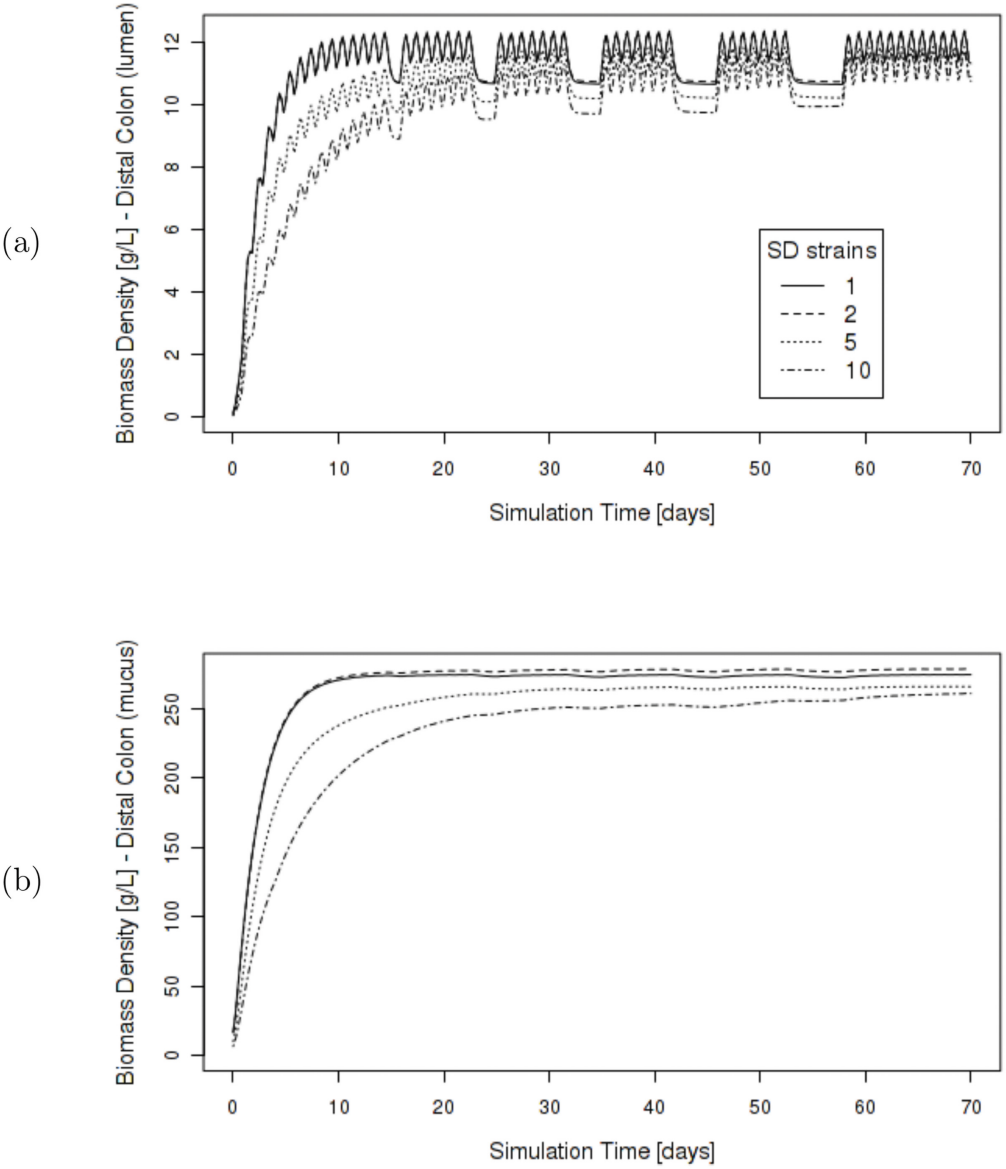
Effect of microbial complexity on stabilizing diet perturbations. (a) Percentage of material that is biomass on colon output (distal gut) associated with the lumen micro-environment. Singular strain representation within the sugar utilizing functional group results in the percent biomass steadily decreasing after repeated zero-diet periods. Multiple strains prove to provide better stability throughout simulation. (b) Percentage of material that is biomass on colon output (distal gut) associated with mucus environment.

Microbial representation has significant effect on coarse colon description during simulations with dietary perturbations (Fig 5). Simulations with single biomass representation within the sugar utilizing functional group are less capable of withstanding repeated perturbations to incoming diet, as the percentage of material in the colon at the distal output of the colon slowly diminishes through the duration of the simulation. This occurs in both the lumen and mucus environments, but is most evident in the plot of mucus composition. In contrast, simulations with 2, 5 or 10 biomass representatives show that the microbial community remains stable through these major swings in diet. The difference between having 2, 5 or 10 representatives within the group is subtle, indicating that the key factor is having at least two functional representatives to maintain activity during substrate scarcity.

#### Microbial Community Perturbation

**Fig 6:**
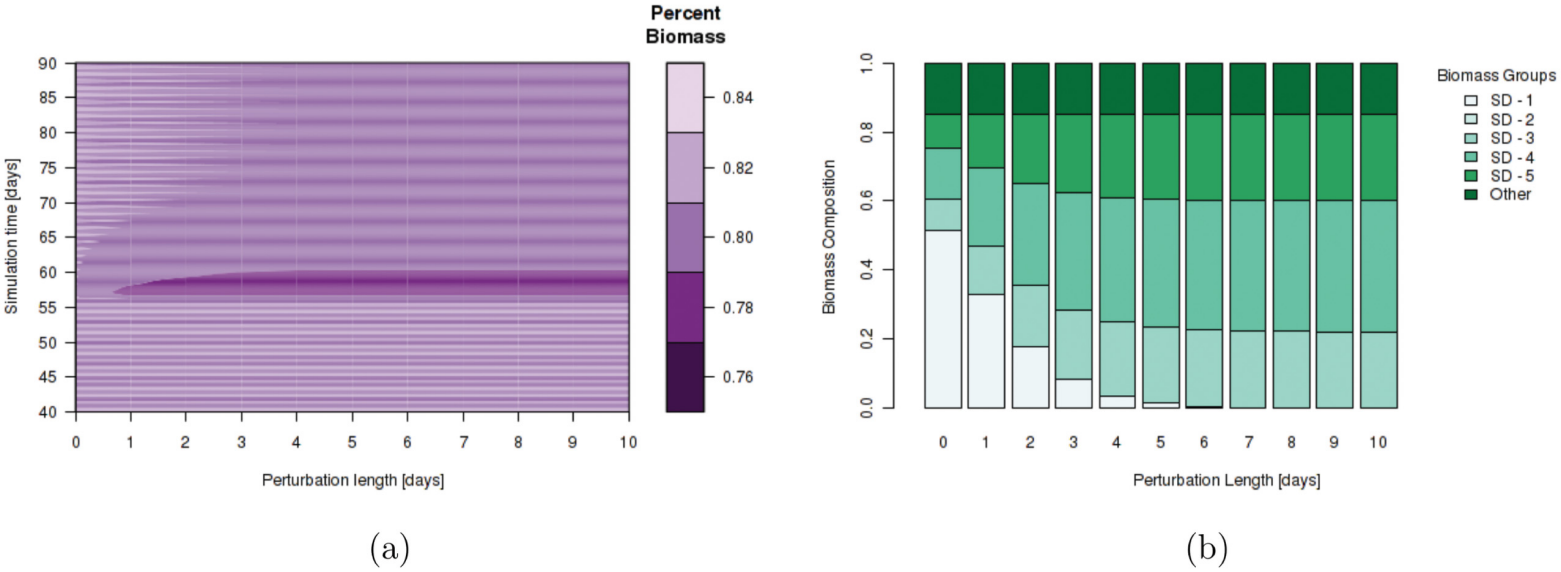
Effect of microbial perturbation on total material composition and community structure. (a) Percent biomass of total material composition at colon output during simulation with perturbations. (b) Composition of microbial community 56 days after end of perturbation period for various perturbation lengths. Perturbation in this scenario is characterized as an increased removal (decay) rate of the dominant sugar utilizing biomass species in the system after 56 days.

Increased microbial representation allows for the colon process model to recover performance during major disruptions to the microbiota community. Fig 6a illustrates the coarse composition (percent total biomass) at the output of the colon as a function of time for given perturbation lengths where perturbation is defined as an increase in decay rate for the major sugar utilizer over the perturbation length. It can be seen that regardless of perturbation length, the system is able to recover to its steady, or *healthy*, state within 4 days. Fig 6b illustrates the detailed microbial community structure 56 days after the perturbation period for the 10 perturbation periods. Even though coarse material composition is recovered independent of perturbation length, the microbial community structure in simulations with perturbations of any length vary significantly. In simulations with perturbation lengths greater than 5 days, the major/removed sugar utilizing biomass representative (SD-1) is never recovered. The sugar utilizing biomass strain that becomes the new major or dominant strain after perturbation is dependent on parameter choice as can be seen in the supplementary animations.

## Discussion

Significant developments regarding microbiota and human interaction have been made with respect to experimental measures and analytical techniques [32], creating an influx of new information and observation. In order to bridge knowledge to understanding, we require mechanistic models, allowing us to extrapolate theory and generate questions. In this paper, we presented a comprehensive framework for mathematical modeling of colon fermentation with preliminary sub-models of digestion, exchange, and convective transport of material through the system. We were able to obtain results with qualitative similarities to those achieved *in vivo* and *in vitro*. With future modifications to the *compuGUT* output measurements, we may have the opportunity to generate results quantitatively comparable to those generated through experiment. With newly developing spatial measurement technology to measure aspects of digestion through the length of the system in live animals, such as the Smart Pill [33, 34], direct testing with experiments that mimic our simulations may be possible. Here we highlight some of the model limitations, as well as potential applications given its current development.

One of the primary simplifications made during model development is the physical representation underlying the model structure—a spatially continuous pipe with fixed-media attached to pipe walls and fluid media convected in a single direction. Traditional *in vitro* experimental systems, as well as previous mathematical models of carbohydrate digestion in the colon [18], employ a three-stage description, attempting to capture the three basic sections of the colon (proximal, transverse and distal). As shown in our assessment of physical reactor representation [22] and confirmed in this study, the output (distal colon) generated through either the three-stage, continuous, or even single-stage model are comparable. However, we have shown that: (i) the material at colon output is not a sufficient proxy of the material in the proximal colon, and that (ii) the characterization of material in the proximal end of the colon is highly dependent on the reactor representation. Due to our model sharing many of the assumptions as the three-stage model, but also having a more refined perspective of the entire colon length, we would assume that the spatially continuous model provides the more likely description of the proximal colon. However, we cannot verify this claim given current experimental procedures. We should also note that the spatially continuous model requires significantly more computing time (≈ 15×), and so one can argue that the three-stage model is a more suitable tool if interest lies primarily in studying the distal colon.

The physiological colon is spatially continuous, but of irregular cross-sectional geometry due to villi and compression [27]. Though our model is capable of providing insights that are more detailed than what has been made available to date from both *in vitro* and *in silico* models, it cannot guarantee complete description of colon physiology. Most notably, our physical representation creates a gross simplification of the exchange processes (transport of materials between lumen-mucus-host). Similarly, we employ a fairly substantial simplification regarding material properties and flow dynamics, which also creates most difficultly in assessing exchange processes. Given our current goals of modeling microbiota behavior in the colon and the top-down structure of our model leading to short-chain fatty acid concentrations not affecting microbial densities, the accuracy with which we model exchange processes is not paramount. That said, short-chain fatty acids produced in the colon are of great importance to the physiological function of the colon [35–37], and so future model extensions that venture into the territory of modeling the gut as oppossed to gut processes should place high importance on modeling exchange.

An approach to better capture physiological accuracy is to refine the convective flux model to segregate peristaltic forces rather than average these forces to a singular expression, and to capture the changing material properties of the digesta passing through the colon. In [38, 39] the authors were able to mathematically describe viscosity changes of *chyme* in a model of digestion in the small intestine. The challenge of implementing such a description in our current framework is the presence of intestinal microbiota. In the small intestine, digestion is a chemical process independent of water availability. In the large intestine, digestion is completed by anaerobes and so the rate of digestion is highly dependent on water availability, strengthening the mathematical coupling of digestion, flow, and exchange processes. Thus incorporating viscosity changes may require an overhaul of how reaction and exchange parameters are defined, but such an overhaul will provide a model much more capable of describing behaviour closer to the true colon.

As noted, we extend the MT-model of carbohydrate digestion by anaerobic microbiota in [18] using an adaptation of a method described in [24]. We found that in simulation of ideal scenarios with zero perturbations that modeling microbial complexity provides no additional value, however, the redundancy introduced by microbial complexity is valuable in maintaining digestion efficacy in non-ideal, or realistic, simulations, consistent with the findings of [24]. Additionally, we find that after a simulated perturbation (increased removal rate of the dominant sugar utilizing biomass strain over a specified length of time) that the microbial community structure can be directed to an alternate *steady state*. Potential health implications of gut flora diversity changes are discussed in [40, 41]. Based on our generic modeling approach, we cannot comment on the health implications of alternate gut flora steady states achieved using our model. Clinically, incomplete recovery of distal gut microbiota to repeated antibiotic perturbations was observed in [42]. Though our modeled perturbation is not directly comparable to the process of repeated antibiotic treatments, the ability of our model to capture a similar phenomenon is a step in the direction towards mechanistic understanding.

At its present state, the computational model is limited to modifications of input dietary fiber, initial conditions, physical parameters (flow rate, volume, mucus production rates) and microbial representations. However, we have yet to fully explore the complete model output space due to its high dimensionality. That is, the model is capable of generating a vast amount of spatial and dynamics data that can be interpreted in a variety of lights. In this paper, we presented perspectives using *coarse material composition*, as well as *microbial community structure*, but rigorous mining and analysis of simulation data could lead to additional insights that we have overlooked.

With model extensions to better describe material/flow properties incorporating the concept of viscosity and muscular peristalsis, and a more complete digestion process including digestion of multiple substrates, i.e. the model of [17], as well as interaction with non-functional/suppressed microbiota, we will be able to simulate an even more diverse variety of scenarios which may be more comparable to those seen in clinical investigation.

## Conclusions

We presented a spatially continuous model of carbohydrate digestion and transport processes in the colon—an extension of the three-stage model described in [18]. Both models provide near identical descriptions of distal colon output under standard physiological simulation conditions. However, output assessed from the proximal colon models of both configurations differ under standard physiological simulation conditions, and the output assessed independent of colon location in both configurations differs in non-standard simulation conditions. This implies that using the distal colon as a proxy for material composition in the proximal colon would be unrealistic. Further assessment of this implication to *in vitro* and *in vivo* experimentation should be conducted.

The extended microbial representation in the description of carbohydrate degradation by intestinal anaerobes provides us the ability to simulate system perturbations. We find that the overall system performance, judging how the anaerobic digestion process proceeds, during a period of distress is tempered by having a diverse microbial community present. However, the composition of the microbial community after a distress/perturbation period is often not the same as it was prior to that period. How the post-distress microbial community affects host-health is an important follow up question.

## Supporting Information

S1 Appendix Mathematical Details. Model construction and numerical treatment. This document details the construction of the underlying mathematical models, and includes important information necessary for the reproduction of the numerical program.
(PDF)
