# Supplementary material for: A Spatially Continuous Model of Carbohydrate Digestion and Transport Processes in the Colon
Source: PLoS One. 2015 Dec 17;10(12):e0145309. doi: 10.1371/journal.pone.0145309 (PMC4683063; doi:10.1371/journal.pone.0145309)
Supplement: S1 Appendix — This document details the construction of the underlying mathematical models, and includes important information necessary for the reproduction of the numerical program. (PDF) [file pone.0145309.s001.pdf]

S1 Appendix. Mathematical Details

1 Overview

The large intestine is the distal most portion of the gastro-intestinal tract. It is responsible for the final absorption of digestive nutrients and preparation of fecal matter for bowel movements [1]. It is an open tube-like organ with muscular walls to aid in the continued transport of eventual waste materials. The walls of the colon are also lubricated with endogenously produced mucus. The colon is often described in three separate locations: the proximal (or ascending), transverse, and distal (or descending) colon. These three locations have differing physical conditions, specifically with regard to the acidity (with locations closer to the proximal end being more acidic than towards the distal end) and the absorption/transportation rates at which substrates are removed from the colon [1]. The colon's biochemical environment makes it a highly suitable habitat to dense communities of microflora. One of the primary functions of the intestinal microflora is to digest chemically indigestible materials (such as dietary fiber). Metabolites generated through this digestion process are absorbed by the gut, and waste material is transported along the length of the colon. Thus, we can think of colon functionality as being defined by three sub-processes with dynamics governed by the interaction of a complex network of microflora, substrates, metabolites and physical forces, in multiple physically and biochemically diverse environments: (i) the digestion of particulate material, (ii) the exchange of soluble

materials between biochemical environments (lumen-mucus-host), and (iii) the convective transport of materials through the length of the colon.

By way of material balance, we can combine the three sub-processes and describe the density of materials in the colon with the following advection-reaction system:

$$\partial_t \mathbf{c} + \partial_x F(\mathbf{c}) = R(\mathbf{c}) + E(\mathbf{c}) \tag{1}$$

where the functions R, F and E can be interpreted as non-linear functions describing the sub-processes of anaerobic digestion, material transport, and component exchange, respectively, and their input, $\mathbf{c}$, is a vector of concentrations [g/L] of all materials considered in the colon-complex. We describe functions R, F and E in detail in the following sections, but present Figure 1 as a schematic representation of the model structure and foundational processes.

1.1 Assumptions

Physiological systems are highly complex, functioning with redundancy, time-variations and interplay with other systems [3]. Rather than model the entire physiology of the colon, we look to capture the integral mechanisms defining the colon-diet-flora system with as little complexity as possible. We introduce the following simplifications for model development:

- **Colon Geometry:** A cross-sectional slice of the colon would display highly irregular geometry, as there exists mucousal folds and villous

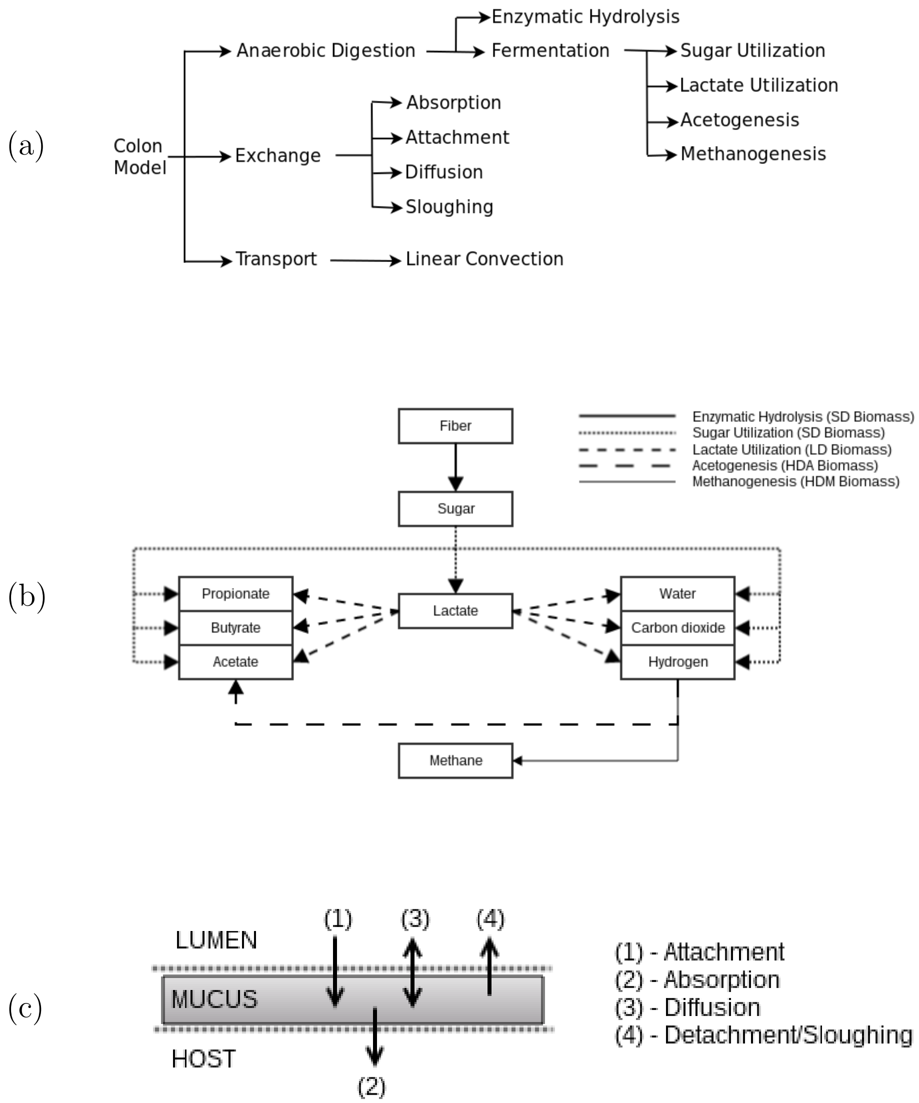

Figure 1: Non-technical overview of compuGUTs underlying mathematical model. (a) Schematic overview of biochemical and physical processes considered in the compuGUT. (b) 5-step model of anaerobic digestion, adapted from [2]. Biomass functional group active in each step indicated in parentheses. (c) Summary of component exchange processes. Material in the lumen environment is transported along the length of the colon where as mucus material is stationary along the length of the colon and only experiences axial transport.

surfaces [4, 5]. For simplicity, the geometry of the large intestine is averaged as a cylindrical tube of constant diameter. Combining this simplification with the knowledge that the length of the colon is significantly larger than its diameter allows us to model the colon as 1-dimensional in space (x-dimension)

- **Material Properties:** To be consistent with our first assumption (1D tube geometry), we assume that the materials in localized portions of the colon (a particular x-value) will be homogeneous (well-mixed) across the cross-sectional area.

- **Mucus Thickness:** Mucus is produced endogenously through out the colon. The rate of this mucus production is constant in all locations. Additionally, we treat this layer as a fixed medium of constant volume, with the volume of the mucus being 10% of the total colon volume.

- **Transit Time:** The effect of peristalsis and additional propulsion mechanisms manifest as an average flow or speed of convective transport. This allows us to approximate convective transport as a first-order flux with constant velocity term.

- **Metabolic Pathways:** The only macromolecules reaching the colon are carbohydrates, and the anaerobic digestion of carbohydrates follows the metabolic pathway described in [2]. This metabolic pathway can be summarized as a five-step process (highlighted in Figure 1b), where

fiber is first hydrolyzed to monomer sugars, and then monomer sugars are fermented by intestinal microflora into various metabolites (lactate, acetate, propionate, butyrate, hydrogen, methane, carbon dioxide, and water) in the parallel processes of sugar utilization, lactate utilization, acetogenesis and methanogenesis. Though there are over 400 species of microbes inhabiting the colon [6], we assume that the total flora in the colon can be sub-divided into four biomass functional groups according by fermentation step. Thus we define flora as either Sugar Fermenters (SD), Lactate Fermenters (LD), Hydrogen Oxidizing Acetogens (HDA), or Hydrogen Oxidizing Methanogens (HDM). Hydrolysis progresses due to enzymes produced by SD flora. These assumptions are adapted directly from the works of [7, 8, 2], and is a familiar approach in most lines of microbial modeling and engineering.

- **Reaction Processes:** Combining the processes involved in metabolism and the natural decay of flora in the system, we can summarize the reaction processes in the flora-diet system as: (1) hydrolysis, (2) glucose utilization, (3) lactate utilization, (4) acetogenesis, (5) methanogenesis, (6) decay of SD flora, (7) decay of LD flora, (8) decay of HDA flora, and (9) decay of HDM flora. The choice of metabolic pathways and subsequent reaction processes is responsible for the overall model problem size, thus a simpler representation of anaerobic digestion would lead to a smaller state-space, and a more involved representation of anaerobic digestion would lead to a larger state space.

We remark that the model assumptions and simplifications can be relaxed on future model iterations as knowledge of functional details continues to grow, but doing so would require the inclusion of additional mathematical and numerical complexities.

1.2 Notation

For organizational convenience, we introduce notation conventions prior to proceeding with the model construction. With the size, complexity and included variability of the mathematical description, the model is more naturally suited for numerical investigation. Accordingly, we follow a computational array/indexing organization scheme, which will also allow for discussion of the software implementation.

1.2.1 Indices

Our primary indices are i, j, and e. Index i indicates particularity within a solution array/parameter grouping. Index j indicates particularity within index i (if needed). Index e, when associated with a model variable or parameter indicate the biochemical environment (lumen or mucus) in which that particular component exists or parameter is used. Index e takes the value 1 if describing a lumen variable, and 2 if identifying a mucus variable. Descriptions of the solution arrays and parameter groupings in which these indices are used is to follow, and will aid in clarity.

1.2.2 Dependent variables

We notate our comprehensive solution array (all dependent variables) by the vector $\mathbf{c}$ - concentration, where $\mathbf{c} = [\mathbf{S}, \mathbf{I}, \mathbf{X}]^T$, and the components of sub-solution arrays $\mathbf{S}$ - soluble substrates/metabolites/compounds, $\mathbf{I}$ - insoluble carbohydrates, and $\mathbf{X}$ - biomass, are defined as follows:

$$S_{e,i_s} \quad \text{where} \quad i_s \in [1,2,3,4,5,6,7,8,9], \tag{2}$$

$$I_{e,i_i} \quad \text{where} \quad i_i \in [1], \tag{3}$$

$$\begin{aligned} X_{e,i_x,j_{i_x}} \quad \text{where} \quad & i_x \in [1,2,3,4], \\ & j_{i_x} \in [1,2,...,n_{i_x}], \end{aligned} \tag{4}$$

and index $e \in [1,2]$ is as previously defined. All dependent variables are concentrations measured in [g/L]. The use of subscripts with indices i and j is to make clear that their values are dependent on the solution array $(\mathbf{S}, \mathbf{I}, \mathbf{X})$ or biomass functional group (in the case of j) being considered. Moving forward, we drop these subscripts for legibility whenever possible but do include them in situations which may otherwise read ambiguously. The use of index j when describing biomass quantities is to identify *a strain or species* within the biomass functional group indexed by i, where the maximum value of j is n_i. That is, $X_{2,3,5}$ would identify the concentration of the 5th *species* of acetogenic biomass ($i = 3$) in the mucus environment ($e = 2$). Details on how strains are defined are to follow in Section 2.1.2. A summary list of

dependent variables, including their mathematical identification and numerical implementation indices is given in Table 1. Referring back to the overall solution array $\mathbf{c}$, we can summarize the overall problem (system) size as:

$$\dim(\mathbf{c}) = \max(e) \times \left(\max(i_s) + \max(i_i) + \sum_{i=1}^{\max(i_x)} \max(j_i) \right), \tag{5}$$

detailing that the problem size is equal to the sum of the maximum number of substrate, fiber, and biomass representations multiplied by the number of environments (lumen and mucus). For the most simple model scenario we present (1 strain per biomass functional group), this would mean a state vector of 28 elements (9 substrates, 1 fiber, 4 biomass functional groups, 1 strain per biomass functional group, 2 environments), and for the most complex scenario that we have tested, a state vector of 100 elements (9 substrates, 1 fiber, 4 biomass functional groups, 10 strain per biomass functional group, 2 environments).

1.2.3 Parameters

The model contains many parameters of similar definition, so standardized notation is used to maintain organization.

There are four primary groups of parameters: yield coefficients (Y), kinetic rates (κ), half-saturation constants and concentrations (K), and exchange rates (γ). Additional physical (lengths, volumes, etc.) and operational (variance, spline constants) parameters exist, but will be described as they are introduced in the text.

Table 1: Summary of dependent variable notation used to describe the colon-complex. C.Index indicates the index value used in numerical implementation, whereas indices i, j and e indicate index values used for mathematical development, as described in Section 1.2.

C.Index	Solution Array	i	j	e	Component
1	S	1		1	Lumen glucose
2		1		2	Mucus glucose
3		2		1	Lumen lactate
4		2		2	Mucus lactate
5		3		1	Lumen hydrogen
6		3		2	Mucus hydrogen
7		4		1	Lumen acetate
8		4		2	Mucus acetate
9		5		1	Lumen propionate
10		5		2	Mucus propionate
11		6		1	Lumen butyrate
12		6		2	Mucus butyrate
13		7		1	Lumen methane
14		7		2	Mucus methane
15		8		1	Lumen carbon dioxide
16		8		2	Mucus carbon dioxide
17		9		1	Lumen water
18		9		2	Mucus water
19	I	1		1	Lumen polysaccharide (fiber)
20		1		2	Mucus polysaccaride (mucin)
$2(10)+1$	X	1	1	1	Lumen sugar utilizing biomass - strain 1
$\vdots$					
$2(10+n_1)$		1	n_1	2	Mucus sugar utilizing biomass - strain n_1
$2(10+n_1)+1$		2	1	1	Lumen lactate utilizing biomass - strain 1
$\vdots$					
$2(10+n_1+n_2)$		2	n_2	2	Mucus lactate utilizing biomass - strain n_2
$2(10+n_1+n_2)+1$		3	1	1	Lumen acetogenic biomass - strain 1
$\vdots$					
$2(10+\sum_i^3 n_i)$		3	n_3	2	Mucus acetogenic biomass - strain n_3
$2(10+\sum_i^3 n_i)+1$		4	1	1	Lumen methanogenic biomass - strain 1
$\vdots$					
$2(10+\sum_i^4 n_i)$		4	n_4	2	Mucus methanogenic biomass - strain n_4

Yield coefficients describe the affects of anaerobic reaction process on the density of system variable. We use the following standard notation:

$$Y_{i_c,p_r,j_{i_x}} \quad \text{where} \quad p_r \in [1,2,3,4,5,6,7,8,9], \\ j_{i_x} \in [1,2,...,n_{i_x}] \tag{6}$$

indicating the yield of system variable i_c consumed or generated in the anaerobic reaction process (see Section 1.1) p_r performed by strain j_{i_x} of biomass functional group i_x. It should be noted that yield coefficients exist for all components of vector $\mathbf{c}$, hence the use of index i_c. That said, not all components are involved in all processes, leading to yield coefficients of zero.

Kinetic parameters specify the maximum rate at which a reaction process indexed by p_r and governed by biomass strain j_{i_x} proceeds, and takes the standard notation:

$$\kappa_{p_r,j_{i_x}} \quad \text{where} \quad p_r \in [1,2,3,4,5,6,7,8,9], \\ j_{i_x} \in [1,2,...,n_{i_x}]. \tag{7}$$

Similarly, many of our considered reaction kinetics have half saturation constants or concentrations, following the standard notation:

$$K_{p_r,j_{i_x}} \quad \text{where} \quad p_r \in [1,2,3,4,5,6,7,8,9], \\ j_{i_x} \in [1,2,...,n_{i_x}]. \tag{8}$$

Lastly, specific rates are used to describe the way material is exchanged between biochemical environments using 4 exchange mechanisms indexed by

p_e, taking the standard notation:

$$\gamma_{i_c,p_e} \quad \text{where} \quad p_e \in [1,2,3,4]. \tag{9}$$

Like yield coefficients, exchange rates exists for all components of the solution array $\boldsymbol{c}$ for each exchange process, which is why we use the index i_c. A complete list of all biochemical reaction (Yield and rate coefficients) and exchange parameters with default values is provided in Tables 2 and 3, and all physical and operation parameters are defined in Tables 4 and 5.

2 Model Development

We construct the mathematical model using material balances to describe how quantities in the colon-complex change with time and space. The result is a system of partial differential equations with functional representations of sub-processes as summarized in (1).

2.1 Anaerobic Digestion

As noted in our primary assumptions, the choice of anaerobic digestion/metabolic pathway is key to determining the size and structure of the mathematical model. Digestion occurs throughout the length of the colon, and in both the lumen and mucus environments. For clarity, we describe our model of anaerobic digestion independent of location and environment.

Table 2: List of default simulation biochemical reaction parameters. **YC**: Yield Coefficient, **SR**: Specific Rate **CR**: Concentration Ratio, **HS**: Half-Saturation. Column one presents the parameter reference number used in the sensitivity analysis. Yield coefficients are derived using stoichiometry balances (Section 2.1). Reaction parameter values adapted from [2]. Yield parameters are described in grams of product per gram of limiting reactant for ease of identification.

**SA ref.**	**Symbol**	**Parameter**	**Value**
	$Y_{1,1}$	YC sugar from fiber	1 g_{su}/g_z
	$Y_{2,2}$	YC lactate from sugar	0.0901 g_{la}/g_{su}
	$Y_{3,2}$	YC hydrogen from sugar	0.00606 g_{H_2}/g_{su}
	$Y_{4,2}$	YC acetate from sugar	0.12121 g_{ac}/g_{su}
	$Y_{5,2}$	YC propionate from sugar	0.14949 g_{pro}/g_{su}
	$Y_{6,2}$	YC butyrate from sugar	0.04444g_{but}/g_{su}
	$Y_{3,3}$	YC hydrogen from lactate	0.00444 g_{H_2}/g_{la}
	$Y_{4,3}$	YC acetate from lactate	0.06667 g_{ac}/g_{la}
	$Y_{5,3}$	YC propionate from lactate	0.16444 g_{pro}/g_{la}
	$Y_{6,3}$	YC butyrate from lactate	0.09778 g_{but}/g_{la}
	$Y_{4,4}$	YC acetate from hydrogen	2.14286 g_{ac}/g_{H_2}
	$Y_{7,5}$	YC methane from hydrogen	0.57143 g_{CH_4}/g_{H_2}
	$Y_{8,2}$	YC carbon dioxide from sugar	0.13333 g_{CO_2}/g_{su}
	$Y_{8,3}$	YC carbon dioxide from lactate	0.14667 g_{CO_2}/g_{la}
	$Y_{8,4}$	YC carbon dioxide from hydrogen (acetogenesis)	-11.000 g_{CO_2}/g_{H_2a}
	$Y_{8,5}$	YC carbon dioxide from hydrogen (methanogenesis)	-9.42857 g_{CO_2}/g_{H_2}
	$Y_{9,2}$	YC aqueous water from sugar	0.12364 g_{H_2O}/g_{su}
	$Y_{9,3}$	YC aqueous water from lactate	0.20000 g_{H_2O}/g_{la}
	$Y_{9,4}$	YC aqueous water from hydrogen (acetogenesis)	6.42857 g_{H_2O}/g_{H_2a}
	$Y_{9,5}$	YC aqueous water from hydrogen (methanogenesis)	6.42857 g_{H_2O}/g_{H_2m}
	$Y_{11,2}$	YC sugar degrading bacteria	0.3424 $g_{X_{su}}/g_{su}$
	$Y_{12,3}$	YC lactate degrading bacteria	0.37667 $g_{X_{la}}/g_{la}$
	$Y_{13,4}$	YC acetogenic bacteria	4.035714 $g_{X_{H_2a}}/g_{H_2}$
	$Y_{14,5}$	YC methanogenic bacteria	4.035714 $g_{X_{H_2m}}/g_{H_2}$
1	κ_1	SR hydrolysis	10.6195 $g_z/g_{X_{su}} \cdot d$
2	κ_2	SR sugar consumption	12.6271 $g_{su}/g_{X_{su}} \cdot d$
3	κ_3	SR lactate consumption	82.1083 $g_{la}/g_{X_{la}} \cdot d$
4	κ_4	SR hydrogen consumption by acetogenic bacteria	1.9263 $g_{H_2}/g_{X_{H_2a}} \cdot d$
5	κ_5	SR hydrogen consumption by methanogenic bacteria	0.3997 $g_{H_2}/g_{X_{H_2m}} \cdot d$
6	K_1	CR (hydrolysis)	0.2654 $g_z/g_{X_{su}}$
7	K_2	HS concentration sugar	0.4684 g_{su}/L
8	K_3	HS concentration lactate	0.5969 g_{la}/L
9	K_4	HS concentration hydrogen (acetogenesis)	0.0034 g_{H_2}/L
10	K_5	HS concentration hydrogen (methanogensis)	3.126×10^{-6} g_{H_2}/L
11-14	κ_{6-9}	SR biomass decay	0.01 d^{-1}

Table 3: Matrix of exchange terms for soluble substrates, polysaccharides and biomass concentrations. Functions E_1,E_2,E_3, and E_4 represent transport from lumen to mucus, from mucus to host, between lumen and mucus, and from mucus to lumen, respectively. The *shape* of exchange parameter γ as a function of colon location (Described in Section 2.2) is shown as a spark figure, with the discrete values in the proximal (P), transverse (T) and distal (D) colon provided in brackets. Exchange parameters are in the units $[d^{-1}]$ except with respect to transport between lumen and mucus (E_3), where the units are [L/d]. Discrete parameter values taken from [2].

**Process, p_e**		E_1 $[L \rightarrow M]$		E_2 $[M \rightarrow H]$		E_3 $[L \leftrightarrow M]$		E_4 $[L \leftarrow M]$	
**Component k**		γ_1	[P/T/D]	γ_2	[P/T/D]	γ_3	[P/T/D]	γ_4	[P/T/D]
1	s_s						[1.6/3.8/6.3]		
2	s_l		[0.88/0.43/2.03]		[12.6]				
3	s_h								
4	s_{ac}		[1.32/0.64/3.05]		[18.9]				
5	s_{pr}		[1.07/0.62/2.47]		[15.32]				
6	s_{bu}		[0.9/0.6/2.49]		[12.88]				
7	s_{ch_4}		[1/0.6/3]		[14]				
8	s_{co_2}		[1/0.6/3]		[14]				
9	s_{h_2o}		[1.6/0.77/3.66]		[1.6]				
10	z								[0.1]
11	b_s		[0.1]						[0.4]
12	b_l		[0.1]						[0.4]
13	b_a		[0.1]						[0.4]
14	b_m		[0.1]						[0.4]
**Kinetic Rate**		$E_1 = \gamma_{1,k} c_{1,k}$		$E_2 = \gamma_{2,k} c_{2,k}$		$E_3 = \frac{\gamma_{3,k}}{V_l}(c_{1,k} - c_{2,k})$		$E_4 = \gamma_{4,k} c_{2,k}$	

Table 4: Physical parameters required for simulation. Parameter values adapted from [2].

Symbol	Parameter	Default Value
L_c	Length of colon [m]	1.524
d_c	Average diameter of the colon [cm]	7.62
L_s	Length of small intestine [m]	6.096
d_s	Average diameter of small intestine [cm]	2.54
V_c	Volume of colon [L]	6.95
V_l	Volume of lumen environment [L]	6.255
V_m	Volume of mucus environment [L]	0.695
$L_{p,t}$	Proximal-Transverse colon length transition percentage	0.14
$L_{t,s}$	Transverse-Distal colon length transition percentage	0.42
q	Average system flow rate [L/d]	7

Table 5: Operation parameters required for simulation. Parameter values adapted from [2].

Symbol	Parameter	Default Value
n_1 (n_{sd})	Number of sugar utilizing biomass representatives	1
n_2 (n_{ld})	Number of lactate utilizing biomass representatives	1
n_3 (n_{hda})	Number of acetogenic biomass representatives	1
n_4 (n_{hdm})	Number of methanogenic biomass representatives	1
σ_b	Variance of biochemical reaction parameters	0.05
σ_p	Variance of exchange parameters	0.0
σ_s	Cubic spline interpolation range (percentage)	0.1
k	Grid Index value	0
N	Number of grid points ($(50)2^k + 1$)	51

2.1.1 MT-Model

A model of anaerobic digestion specific to the environmental conditions of the human colon was developed in [2], simplifying the Anaerobic Digestion Model No. 1 (ADM1) system described in [8] to only consider carbohydrate particulate waste (as opposed to including proteins and lipids as well), and employ lumen and mucus *environments* to describe the colons physical structure. We refer to this model as the MT-model of carbohydrate digestion. The resulting model describes anaerobic digestion in two processes (enzymatic hydrolysis and fermentation) consisting of five metabolic steps, all of which are driven by the presence of microflora, and the natural decay of biomaterial from the system.

Enzymatic Hydrolysis: Enzymatic hydrolysis is the degradation of polysaccharides into simple monosaccharides in the presence of enzymes produced by sugar utilizing biomass. The complete process of enzymatic hydrolysis is quite complex and is composed of a large number of intermediate steps [9, 10]; however, mathematical models of the rate of hydrolysis are often simplified to statements of first-order based on observation and empirical data [11]. In [2], the authors suggest modeling hydrolysis by *Contois* kinetics, as equations of this form are well adapted for modeling a wide range of substrate-biomass scenarios [12]. As such, we model the rate of hydrolysis (ϕ_1) as follows:

$$\phi_1 = \frac{\kappa_1 I X_1}{I + K_1 X_1}, \tag{10}$$

where variables and parameters are as previously defined.

Fermentation: Fermentation is the process of converting simple sugars to short-chain fatty acids, simple compounds and gases. The steps within fermentation, which occur both sequentially and in parallel, create time sensitivities and model stiffness. Additionally, the rates at which these steps occur is a product of microflora concentration and substrate/metabolite availability. The rates for (i) glucose utilization, (ii) lactate utilization, (iii) acetogenesis, and (iv) methanogenesis, are all modeled using *Monod* kinetics, as:

$$\phi_f = \frac{\kappa S X}{K + S} I_{pH}, \tag{11}$$

where S is the concentration of substrate utilized by biomass X in the completion of a particular fermentation step and I_{pH} is a rate inhibition term due to acidity. Most fermentation steps are not pH inhibited, thus $I_{pH} = 1$.The rate of methanogenesis is inhibited as follows:

$$I_{pH} = \begin{cases} exp(-3\left(\frac{pH - pH_u}{pH_u - pH_l}\right)^2) & \text{if } pH < pH_u, \\ 1 & \text{if } pH \geq pH_u, \end{cases} \tag{12}$$

where pH_u and pH_l are upper and lower pH limits that are dependent on colon location [2, 8].

Natural Decay: The effects of age and damage do apply to microbial systems [13]. This natural decay is included as:

$$\phi_d = \kappa X, \tag{13}$$

where X is a biomass concentration and κ is the specific rate of decay for that particular biomass strain.

Derivation of Yield Coefficients: Each process in the fermentation of simple sugars to SCFAs can be expressed by a balanced chemical equation describing the change from reactants to products. For example, Glucose Fermentation can be described by:

$$\begin{aligned} 11C_6H_{12}O_6 + 6NH_3 \;\; \rightarrow \;\; & 2CH_3CHOHCOOH + 4CH_3COOH \\ & +4CH_3CH_2COOH + CH_3CH_2CH_2COOH \\ & +6H_2 + 6CO_2 + 18H_2O + 6C_5H_7O_2N, \end{aligned}$$

where 11 moles of glucose and 6 moles of ammonia create 2 moles of lactate, 4 moles of acetate, 4 moles of propionate, a mole of butyrate, 6 moles of hydrogen, 6 moles of carbon dioxide, 18 moles of water and 6 moles of biomass, respectively. Biomass involved in glucose utilization is referred to as sugar fermenting or sugar utilizing biomass. The chemical formula for biomass, $C_5H_7O_2N$, is an approximation adapted directly from [8]. Complete chemical balances are provided in Muñoz-Tamayo et. al [2]. For ease of analysis, Tables 6-9 are presented in place of chemical formula to describe the complete reactions associated with fermentation.

Yield coefficients for each product in each reaction process are derived using the mass basis of the process limiting reactant. Process limiting

reactants are identified using boldface in each of the respective tables. For example, the yield of propionate (product) from lactate (reactant) during lactate fermentation is calculated as:

$$\begin{aligned} Y_{5,3} &= \frac{\text{Mass Propionate}}{-\text{Mass Lactate}} \\ &= \frac{\text{Mol. Pro} \times \text{MM Pro}}{-\text{Mol. Lac} \times \text{MM Lac}} = \frac{2 \times 74}{-(-10) \times 90} \approx 0.16444 \end{aligned}$$

where values for stoichiometric coefficients and molar mass are provided in Table 7, and the indices 5 and 3 correspond with the Peterson Matrix shown as Table 10.

Using the described rate equations and derived yield coefficients, the time evolution of material c_i in the resulting *reaction terms* can be written as a set of differential equations in the form:

$$R(c_i) = \dot{c}_i = \sum_{j=1}^{9} Y_{i,j}\phi_j, \tag{14}$$

where variables, processes and indices are as defined in the previous sections and correspond with the Peterson Matrix in Table 10. Analysis of the mass conservation of these reaction terms follows in Section 4.3.

2.1.2 *e*MT-Model

In [14], the authors extend the ADM1 model to simulate *strains* of biomass within a biomass functional group. These *strains* can be identified within a group based on their specified biochemical reaction parameters. We adapt this idea to extend the MT-model of [2] to consider multiple strains as well,

Table 6: Derived yield coefficients for components involved in fermentation step 1: Glucose Utilization.

Index i	Material	Mol. Count	Mol. Mass [g/mol]	Mass [g]	Yield Coefficient [g/g]
1	**glucose**	-11	180	-1980	-1
	ammonia[a]	-6	17	-102	-0.05152
2	lactate	2	90	180	0.09091
3	hydrogen	6	2	12	0.00606
4	acetate	4	60	240	0.12121
5	propionate	4	74	296	0.14949
6	butyrate	1	88	88	0.04444
7	methane	0	16	0	0
8	carbon dioxide	6	44	264	0.13333
9	water	18	18	324	0.12364
10	Fiber	-	-	-	0
11	SD Biomass	6	113	678	0.3424
12	LD Biomass	0	113	0	0
13	HDA Biomass	0	113	0	0
14	HDM Biomass	0	113	0	0
			Sum:	0	4e-5

a - ammonia sources assumed to be readily available and are not formally tracked in mathematical model.

Table 7: Derived yield coefficients for components involved in fermentation step 2: Lactate Utilization.

Index i	Material	Mol. Count	Mol. Mass [g/mol]	Mass [g]	Yield Coefficient [g/g]
1	glucose	0	180	0	0
	ammonia[a]	-3	17	-51	-0.056667
2	**lactate**	-10	90	-900	-1
3	hydrogen	2	2	4	0.00444
4	acetate	1	60	60	0.06667
5	propionate	2	74	148	0.16444
6	butyrate	1	88	88	0.09778
7	methane	0	16	0	0
8	carbon dioxide	3	44	132	0.14667
9	water	10	18	180	0.20000
10	Fiber	-	-	-	0
11	SD Biomass	0	113	0	0
12	LD Biomass	3	113	339	0.37667
13	HDA Biomass	0	113	0	0
14	HDM Biomass	0	113	0	0
			Sum:	0	3e-6

a - ammonia sources assumed to be readily available and are not formally tracked in mathematical model.

Table 8: Derived yield coefficients for components involved in fermentation step 3: Hydrogen Utilizing Acetogenesis.

Index i	Material	Mol. Count	Mol. Mass [g/mol]	Mass [g]	Yield Coefficient [g/g]
1	glucose	0	180	0	0
	ammonia[a]	-1	17	-17	-0.60714
2	lactate	0	90	0	0
3	**hydrogen**	-14	2	-28	-1
4	acetate	1	60	60	2.14286
5	propionate	0	74	0	0
6	butyrate	0	88	0	0
7	methane	0	16	0	0
8	carbon dioxide	-7	44	-308	-11.000
9	water	10	18	180	6.42857
10	Fiber	-	-	-	0
11	SD Biomass	0	113	0	0
12	LD Biomass	0	113	0	0
13	HDA Biomass	1	113	113	4.03571
14	HDM Biomass	0	113	0	0
			Sum:	0	0

a - ammonia sources assumed to be readily available and are not formally tracked in mathematical model.

Table 9: Derived yield coefficients for components involved in fermentation step 4: Hydrogen Utilizing Methanogenesis.

Index i	Material	Mol. Count	Mol. Mass [g/mol]	Mass [g]	Yield Coefficient [g/g]
1	glucose	0	180	0	0
	ammonia[a]	-1	17	-17	-0.60714
2	lactate	0	90	0	0
3	**hydrogen**	-14	2	-28	-1
4	acetate	0	60	0	0
5	propionate	0	74	0	0
6	butyrate	0	88	0	0
7	methane	1	16	16	0.57143
8	carbon dioxide	-6	44	-264	-9.42857
9	water	10	18	180	6.42857
10	Fiber	-	-	-	0
11	SD Biomass	0	113	0	0
12	LD Biomass	0	113	0	0
13	HDA Biomass	0	113	0	0
14	HDM Biomass	1	113	113	4.035714
			Sum:	0	4e-6

a - ammonia sources assumed to be readily available and are not formally tracked in mathematical model.

Table 10: Peterson Matrix of biochemical/metabolic reaction terms for soluble substrates, polysaccharide carbohydrates and biomass concentrations without biomass strain refinement. Adapted from [2].

For soluble components

	**Component i**	1	2	3	4	5	6	7	8	9	**Kinetic Rate**
p_r	**Process**	S_1	S_2	S_3	S_4	S_5	S_6	S_7	S_8	S_9	
1	Hydrolysis	$Y_{1,1}$									$\phi_1(\boldsymbol{c})$
2	Glucose utilization	-1	$Y_{2,2}$	$Y_{3,2}$	$Y_{4,2}$	$Y_{5,2}$	$Y_{6,2}$		$Y_{8,2}$	$Y_{9,2}$	$\phi_2(\boldsymbol{c})$
3	Lactate utilization		-1	$Y_{3,3}$	$Y_{4,3}$	$Y_{5,3}$	$Y_{6,3}$		$Y_{8,3}$	$Y_{9,3}$	$\phi_3(\boldsymbol{c})$
4	Homoacetogenesis			-1	$Y_{4,4}$				$Y_{8,4}$	$Y_{9,4}$	$\phi_4(\boldsymbol{c})$
5	Methanogenesis			-1				$Y_{7,5}$	$Y_{8,5}$	$Y_{9,5}$	$\phi_5(\boldsymbol{c})$

For particulate components

	**Component i**	10	11	12	13	14	**Kinetic Rate**
j	**Process**	I_1	X_1	X_2	X_3	X_4	
1	Hydrolysis	-1					$\phi_1(\boldsymbol{c}) = \kappa_1 \frac{I_1 X_1}{K_1 X_1 + I_1}$
2	Glucose utilization		$Y_{11,2}$				$\phi_2(\boldsymbol{c}) = \kappa_2 \frac{S_1 X_1}{K_2 + S_1}$
3	Lactate utilization			$Y_{12,3}$			$\phi_3(\boldsymbol{c}) = \kappa_3 \frac{S_2 X_2}{K_3 + s_2}$
4	Homoacetogenesis				$Y_{13,4}$		$\phi_4(\boldsymbol{c}) = \kappa_4 \frac{S_3 X_3}{K_3 + S_3}$
5	Methanogenesis					$Y_{14,5}$	$\phi_5(\boldsymbol{c}) = \kappa_5 \frac{S_3 X_4}{K_5 + S_3} I_{pH}$ with $I_{pH} = \begin{cases} exp(-3(\frac{pH - pH_U}{pH_U - pH_L})^2) & \text{if } pH < pH_U, \\ 1 & \text{if } pH \geq pH_U \end{cases}$
6	Decay of X_1		-1				$\phi_6(\boldsymbol{c}) = \kappa_{6,1} X_1$
7	Decay of X_2			-1			$\phi_7(\boldsymbol{c}) = \kappa_{7,1} X_2$
8	Decay of X_3				-1		$\phi_8(\boldsymbol{c}) = \kappa_{8,1} X_3$
9	Decay of X_4					-1	$\phi_9(\boldsymbol{c}) = \kappa_{9,1} X_4$

herein referred to as the eMT-model of carbohydrate digestion. Biochemical parameters for biomass within a group were generated as follows:

$$P_{i,j} = \mathcal{N}(P_i, \sigma), \tag{15}$$

where $P_{i,j}$ is a biochemical reaction parameters (maximum specific growth rate, half-saturation concentration) for the jth strain of biomass functional group i, chosen randomly from the set of values normally distributed around P_i, the default/set value for the parameter assuming single strain representation, with standard deviation as indicated by σ.

This microbial representation extension can be applied naturally in rate models of fermentation (11) and biomaterial decay (13) as previously defined, as each biomass representative within a functional group has its particular parameter set. However, enzymatic hydrolysis of fiber described by contois kinetics must be modeled as followed:

$$\phi_1 = \frac{I \sum_{j=1}^{n_1} \kappa_{1,j} X_{1,j}}{I + \sum_{j=1}^{n_1} K_{1,j} X_{1,j}}, \tag{16}$$

where indices, variables and parameters are as defined previously.

2.2 Component Exchange

The MT-model of [2] also considered separate biochemical environments, the lumen and mucus. Exchange of material $\mathbf{c}$ occurs between these layers both as active (attachment, absorption, detachment) and passive (diffusion) transport. These exchange terms are all linear and vary based on their directionality.

Attachment (lumen → mucus): The active transport of material from the lumen compartment to the mucus compartment. Included materials are lactate, acetate, propionate, butyrate, methane, carbon dioxide, water, and biomass functional groups. This process is modeled as:

$$E_{i,2} = \gamma_{1,i} c_{1,i} \tag{17}$$

Absorption (mucus → host): The active removal of material from the mucus compartment by the body (lactate, acetate, propionate, butyrate, water) or removal as gas (hydrogen, carbon dioxide). This process is modeled as:

$$E_{i,1} = \gamma_{2,i} c_{2,i} \tag{18}$$

Diffusion (mucus ↔ lumen): The passive transport of material between lumen and mucus compartments. Only sugar undergoes diffusive transport. This process is modeled as:

$$E_{i,3} = \frac{\gamma_{3,i}}{V_l}(c_{1,i} - c_{2,i}) \tag{19}$$

Sloughing/Detachment (mucus → lumen): The active removal f material from the mucus back into the lumen. Materials involved in sloughing include particular fiber and biomass functional groups. This process is modeled as:

$$E_{i,4} = \gamma_{4,i} c_{2,i} \tag{20}$$

The rate of exchange varies from location to location along the length of the colon. Experimental approximations for these exchange rates are taken for the coarsely defined locations of the colon (proximal, transverse, distal). The MT-model applied in [2] considers a 3-stage reactor system physical representation analogous to commonly used *in vitro* systems [15], allowing for easy adaptation of experimentally derived exchange parameter approximations. To model the colon as a continuous system, we interpolate exchange parameters as a function of location x by constructing natural cubic splines approximating parameters as a function of length along the colon, using the algorithm described in [16]. We define transition points and regions, outside of which parameters are treated as they would be discretely. For example, we determine the central transition points to be 14% and 42% along the length of the colon, from proximal to transverse and then transverse to distal, respectively, based on approximate colon dimensions [5], and the region of transition to be 10% (as to prevent overlap of regions). This means that 0-4%, 24-32% and 52-100% inclusive along the length of the colon will take the strict parameters associated with discrete proximal, transverse and distal colons, respectively, while the regions of 4-24%, and 32-52% exclusive will transition between the discrete bounds using the cubic approximation. By constructing these spline functions, we emphasize the lack of obvious representation of physiological colon parameters as a function of space due to unavailability of spatially continuous data.

2.3 Transport

As stated previously, we assume that all forces involved in peristaltic movement can be captured in a single *average* flow rate term, which translates to a single convective velocity term

$$F(\mathbf{c}) = \bar{v}\mathbf{c}, \tag{21}$$

where the convective velocity $\bar{v}$ is approximated as:

$$\bar{v} = \begin{cases} 0.001\frac{q}{\pi r^2} & \text{for lumen components} \\ 0 & \text{for mucus components} \end{cases} \tag{22}$$

where q is the average flow rate [L/d] (back-calculated using mean transit times), r is the cross-sectional radius [m], and 0.001 is the metric conversion from litres to cubic meters. As noted in the model assumptions, we treat the colon as a tube with constant cross-sectional radius, meaning the convective velocity is not a function of location x. And so the full model with convective flux evaluated as a velocity term would take the form:

$$\partial_t \boldsymbol{c} + \bar{v}\partial_x \boldsymbol{c} = R(\boldsymbol{c}) + E(\boldsymbol{c}) \tag{23}$$

We expect that a simple *average flow rate*-type approximation will be suitable when simulating the behaviour of colons exhibiting healthy transit times, implicitly assuming well-mixed material and subsequently equal probability exchange. However, the assumptions of well-mixed material should naturally deteriorate as we move along the colon and the viscosity of digesta increases. Describing the physics of these viscosity changes is a current work in progress.

2.4 Endogenous Processes

In our model, we primarily focus on dietary materials and their by-products; effectively disconnecting the colon from other physiological systems. This is seen in the way we account for SCFA absorption as a simple removal term rather than attempting to track its behavior in the body. We do, however, include a description of endogenous mucus production as it is an important stabilizing nutrient source for intestinal microflora. We model the rate of endogenous mucus production Λ as:

$$\Lambda = \Gamma \left(1 - \frac{I_2}{I_M}\right), \tag{24}$$

where I_2 is the fiber of polysaccharides in the mucus environment, Γ is the maximum endogenous mucus production rate [g/Ld], and I_M is the maximum/critical density of fiber in the mucus environment. Including further endogenous processes, namely transport of material from the blood stream into the colon, is a potential model extension.

3 Complete Model

The complete model can be formulated by combining the previously described reaction, exchange and flow processes. To avoid any ambiguity, we write out all partial differential equations that compose the model.

Lumen Components:

Sugar ($S_{1,1}$):

$$
\begin{aligned}
\partial_t S_{1,1} + \bar{v}_l \partial_x S_{1,1} = \quad & Y_{1,1} \frac{I_{1,1} \sum_j^{n_1} \kappa_{1,j} X_{1,1,j}}{\left(\sum_j^{n_1} K_{1,j} X_{1,1,j}\right) + I_{1,1}} && \text{hydrolysis} \\
& - \sum_j^{n_1} \frac{\kappa_{2,j} S_{1,1} X_{1,1,j}}{K_{2,j} + S_{1,1}} && \text{sugar utilization} \\
& - \frac{\gamma_{3,1}}{V_l} (S_{1,1} - S_{2,1}) && \text{diffusion}
\end{aligned}
$$

Lactate ($S_{1,2}$):

$$
\begin{aligned}
\partial_t S_{1,2} + \bar{v}_l \partial_x S_{1,2} = \quad & Y_{2,2} \sum_j^{n_1} \frac{\kappa_{2,j} S_{1,1} X_{1,1,j}}{K_{2,n_1} + S_{1,1}} && \text{sugar utilization} \\
& - \sum_j^{n_2} \frac{\kappa_{3,j} S_{1,2} X_{1,2,j}}{K_{3,j} + S_{1,2}} && \text{lactate utilization} \\
& - \gamma_{1,2} S_{1,2} && \text{attachment}
\end{aligned}
$$

Hydrogen ($S_{1,3}$):

$$
\begin{aligned}
\partial_t S_{1,3} + \bar{v}_l \partial_x S_{1,3} = \quad & Y_{3,2} \sum_{n_1}^{N_1} \frac{\kappa_{2,n_1} S_{1,1} X_{1,1_{n_1}}}{K_{2,n_1} + S_{1,1}} && \text{sugar utilization} \\
& + Y_{3,3} \sum_{n_2}^{N_2} \frac{\kappa_{3,n_2} S_{1,2} X_{1,2_{n_2}}}{K_{3,n_2} + S_{1,2}} && \text{lactate utilization} \\
& - \sum_{n_3}^{N_3} \frac{\kappa_{4,n_3} S_{1,3} X_{1,3_{n_3}}}{K_{4,n_3} + S_{1,3}} && \text{acetogenesis} \\
& - \sum_{n_3}^{N_3} \frac{\kappa_{5,n_3} S_{1,3} X_{1,4_{n_4}}}{K_{5,n_4} + S_{1,3}} I_{pH}(x) && \text{methanogenesis}
\end{aligned}
$$

Acetate ($S_{1,4}$):

$$\begin{aligned}
\partial_t S_{1,4} + \bar{v}_l \partial_x S_{1,4} = {} & Y_{4,2} \sum_{n_1}^{N_1} \frac{\kappa_{2,n_1} S_{1,1} X_{1,1_{n_1}}}{K_{2,n_1} + S_{1,1}} && \text{sugar utilization} \\
& + Y_{4,3} \sum_{n_2}^{N_2} \frac{\kappa_{3,n_2} S_{1,2} X_{1,2_{n_2}}}{K_{3,n_2} + S_{1,2}} && \text{lactate utilization} \\
& + Y_{4,4} \sum_{n_3}^{N_3} \frac{\kappa_{4,n_3} S_{1,3} X_{1,3_{n_3}}}{K_{4,n_3} + S_{1,3}} && \text{acetogenesis} \\
& - \gamma_{1,4} S_{1,4} && \text{attachment}
\end{aligned}$$

Propionate ($S_{1,5}$):

$$\begin{aligned}
\partial_t S_{1,5} + \bar{v}_l \partial_x S_{1,5} = {} & Y_{5,2} \sum_{n_1}^{N_1} \frac{\kappa_{2,n_1} S_{1,1} X_{1,1_{n_1}}}{K_{2,n_1} + S_{1,1}} && \text{sugar utilization} \\
& + Y_{5,3} \sum_{n_2}^{N_2} \frac{\kappa_{3,n_2} S_{1,2} X_{1,2_{n_2}}}{K_{3,n_l} + S_{1,2}} && \text{lactate utilization} \\
& - \gamma_{1,5} S_{1,5} && \text{attachment}
\end{aligned}$$

Butyrate ($S_{1,6}$):

$$\begin{aligned}
\partial_t S_{1,6} + \bar{v}_l \partial_x S_{1,6} = {} & Y_{6,2} \sum_{n_1}^{N_1} \frac{\kappa_{2,n_1} S_{1,1} X_{1,1_{n_1}}}{K_{2,n_1} + S_{1,1}} && \text{sugar utilization} \\
& + Y_{6,3} \sum_{n_2}^{N_2} \frac{\kappa_{3,n_2} S_{1,2} X_{1,2_{n_2}}}{K_{3,n_2} + S_{1,2}} && \text{lactate utilization} \\
& - \gamma_{1,6} S_{1,6} && \text{attachment}
\end{aligned}$$

Methane ($S_{1,7}$):

$$\begin{aligned}
\partial_t S_{1,7} + \bar{v}_l \partial_x S_{1,7} = {} & Y_{7,5} \sum_{n_4}^{N_4} \frac{\kappa_{5,n_2} S_{1,3} X_{1,4_{n_4}}}{K_{5,n_4} + S_{1,3}} I_{pH}(x) && \text{methanogenesis} \\
& - \gamma_{1,7} S_{1,7} && \text{attachment}
\end{aligned}$$

Carbon dioxide ($S_{1,8}$):

$$\begin{aligned}
\partial_t S_{1,8} + \bar{v}_l \partial_x S_{1,8} = \quad & Y_{8,2} \sum_{n_1}^{N_1} \frac{\kappa_{2,n_1} S_{1,1} X_{1,1_{n_1}}}{K_{2,n_1} + S_{1,1}} && \text{sugar utilization} \\
& + Y_{8,3} \sum_{n_2}^{N_2} \frac{\kappa_{3,n_2} S_{1,2} X_{1,2_{n_2}}}{K_{3,n_2} + S_{1,2}} && \text{lactate utilization} \\
& + Y_{8,4} \sum_{n_3}^{N_3} \frac{\kappa_{4,n_3} S_{1,3} X_{1,3_{n_3}}}{K_{4,n_3} + S_{1,3}} && \text{acetogenesis} \\
& + Y_{8,5} \sum_{n_4}^{N_4} \frac{\kappa_{5,n_4} S_{1,3} X_{1,4_{n_4}}}{K_{5,n_4} + S_{1,3}} I_{pH}(x) && \text{methanogenesis} \\
& - \gamma_{1,8} S_{1,8} && \text{attachment}
\end{aligned}$$

Water ($S_{1,9}$):

$$\begin{aligned}
\partial_t S_{1,9} + \bar{v}_l \partial_x S_{1,9} = \quad & Y_{9,2} \sum_{n_1}^{N_1} \frac{\kappa_{2,n_1} S_{1,1} X_{1,1_{n_1}}}{K_{2,n_1} + S_{1,1}} && \text{sugar utilization} \\
& + Y_{9,3} \sum_{n_2}^{N_2} \frac{\kappa_{3,n_2} S_{1,2} X_{1,2_{n_2}}}{K_{3,n_2} + S_{1,2}} && \text{lactate utilization} \\
& + Y_{9,4} \sum_{n_3}^{N_3} \frac{\kappa_{4,n_3} S_{1,3} X_{1,3_{n_3}}}{K_{4,n_3} + S_{1,3}} && \text{acetogenesis} \\
& + Y_{9,5} \sum_{n_4}^{N_4} \frac{\kappa_{5,n_4} S_{1,3} X_{1,4_{n_4}}}{K_{5,n_4} + S_{1,3}} I_{pH}(x) && \text{methanogenesis} \\
& - \gamma_{1,9} S_{1,9} && \text{attachment}
\end{aligned}$$

Fiber ($I_{1,1}$):

$$\begin{aligned}
\partial_t I_{1,1} + \bar{v}_l \partial_x I_{1,1} = & - \frac{I_{1,1} \sum_{n_1}^{N_1} \kappa_{1,n_1} Y_{1,1,n_1} X_{1,n_1}}{\left(\sum_{n_1}^{N_1} K_{1,n_1} X_{1,1_{n_1}} \right) + I_{1,1}} && \text{hydrolysis} \\
& + \left(\frac{V_m}{V_l} \right) \gamma_{4,10} I_{2,1} && \text{sloughing}
\end{aligned}$$

Sugar Degraders ($X_{1,1,n_1}$):

$$\begin{aligned}
\forall n_1 \leq N_1 : \partial_t X_{1,1_{n_1}} + \bar{v}_l \partial_x X_{1,1_{n_1}} = \;& Y_{11,2} \frac{\kappa_{2,n_1} S_{1,1} X_{1,1_{n_1}}}{K_{2,n_1} + S_{1,1}} && \text{sugar utilization} \\
& - \gamma_{1,11_{n_1}} X_{1,1_{n_s}} && \text{attachment} \\
& + \left(\frac{V_m}{V_l}\right) \gamma_{4,11_{n_1}} X_{2,11_{n_1}} && \text{sloughing} \\
& - \kappa_{6,n_1} X_{1,1_{n_s}} && \text{decay}
\end{aligned}$$

Lactate Degraders ($X_{1,2,n_2}$):

$$\begin{aligned}
\forall n_2 \leq N_2 : \partial_t X_{1,2_{n_2}} + \bar{v}_l \partial_x X_{1,2_{n_2}} = \;& Y_{12,3} \frac{\kappa_{3,n_2} S_{1,2} X_{1,2_{n_2}}}{K_{3,n_2} + S_{1,2}} && \text{lactate utilization} \\
& - \gamma_{1,12_{n_2}} X_{1,2_{n_l}} && \text{attachment} \\
& + \left(\frac{V_m}{V_l}\right) \gamma_{4,12_{n_2}} X_{2,2_{n_2}} && \text{sloughing} \\
& - \kappa_{7,n_2} X_{1,2_{n_2}} && \text{decay}
\end{aligned}$$

Hydrogen Degrading Acetogens ($X_{1,3,n_3}$):

$$\begin{aligned}
\forall n_3 \leq N_3 : \partial_t X_{1,3_{n_3}} + \bar{v}_l \partial_x X_{1,3_{n_3}} = \;& Y_{13,4} \frac{\kappa_{4,n_3} S_{1,3} X_{1,3_{n_3}}}{K_{4,n_4} + S_{1,3}} && \text{acetogenesis} \\
& - \gamma_{1,13_{n_3}} X_{1,3_{n_3}} && \text{attachment} \\
& + \left(\frac{V_m}{V_l}\right) \gamma_{4,13_{n_3}} X_{2,3_{n_3}} && \text{sloughing} \\
& - \kappa_{8,n_3} X_{1,3_{n_3}} && \text{decay}
\end{aligned}$$

Hydrogen Degrading Methanogens ($X_{1,4,n_4}$):

$$\begin{aligned}
\forall n_4 \leq N_4 : \partial_t X_{1,4_{n_4}} + \bar{v}_l \partial_x X_{1,4_{n_4}} = \;& Y_{14,5} \frac{\kappa_{5,n_4} S_{1,3} X_{1,4_{n_4}}}{K_{5,n_4} + S_{1,3}} && \text{methanogenesis} \\
& - \gamma_{1,14_{n_4}} X_{1,4_{n_4}} && \text{attachment} \\
& + \left(\frac{V_m}{V_l}\right) \gamma_{4,14_{n_4}} X_{2,4_{n_4}} && \text{sloughing} \\
& - \kappa_{9,n_4} X_{1,4_{n_4}} && \text{decay}
\end{aligned}$$

Mucus Components:

Sugar ($S_{2,1}$)**:**

$$
\begin{aligned}
\partial_t S_{2,1} = \quad & Y_{1,1} \frac{I_{2,1} \sum_{n_1}^{N_1} \kappa_{1,n_1} X_{2,n_1}}{\left(\sum_{n_1}^{N_1} K_{1,n_1} X_{2,1_{n_1}} \right) + I_{2,1}} && \text{hydrolysis} \\
& - \sum_{n_1}^{N_1} \frac{\kappa_{2,n_1} S_{2,1} X_{2,1_{n_1}}}{K_{2,n_1} + S_{2,1}} && \text{sugar utilization} \\
& + \frac{\gamma_{3,1}}{V_m} (S_{1,1} - S_{2,1}) && \text{diffusion}
\end{aligned}
$$

Lactate ($S_{2,2}$)**:**

$$
\begin{aligned}
\partial_t S_{2,2} = \quad & Y_{2,2} \sum_{n_1}^{N_1} \frac{\kappa_{2,n_1} S_{2,1} X_{2,1_{n_1}}}{K_{2,n_1} + S_{2,1}} && \text{sugar utilization} \\
& - \sum_{n_2}^{N_2} \frac{\kappa_{3,n_2} S_{2,2} X_{2,2_{n_2}}}{K_{3,n_2} + S_{2,2}} && \text{lactate utilization} \\
& + \left(\frac{V_l}{V_m} \right) \gamma_{1,2} S_{1,2} && \text{attachment} \\
& - \gamma_{2,2} S_{2,2} && \text{absorption}
\end{aligned}
$$

Hydrogen ($S_{2,3}$)**:**

$$
\begin{aligned}
\partial_t S_{2,3} = \quad & Y_{3,2} \sum_{n_1}^{N_1} \frac{\kappa_{2,n_1} S_{2,1} X_{2,1_{n_1}}}{K_{2,n_1} + S_{2,1}} && \text{sugar utilization} \\
& + Y_{3,3} \sum_{n_2}^{N_2} \frac{\kappa_{3,n_2} S_{2,2} X_{2,2_{n_2}}}{K_{3,n_2} + S_{2,2}} && \text{lactate utilization} \\
& - \sum_{n_3}^{N_3} \frac{\kappa_{4,n_3} S_{2,3} X_{2,3_{n_3}}}{K_{4,n_3} + S_{2,3}} && \text{acetogenesis} \\
& - \sum_{n_3}^{N_3} \frac{\kappa_{5,n_3} S_{2,3} X_{2,4_{n_4}}}{K_{5,n_4} + S_{2,3}} I_{pH}(x) && \text{methanogenesis}
\end{aligned}
$$

Acetate ($S_{2,4}$):

$$\begin{aligned}
\partial_t S_{2,4} = \quad & Y_{4,2} \sum_{n_1}^{N_1} \frac{\kappa_{2,n_1} S_{2,1} X_{2,1_{n_1}}}{K_{2,n_1} + S_{2,1}} && \text{sugar utilization} \\
& + Y_{4,3} \sum_{n_2}^{N_2} \frac{\kappa_{3,n_2} S_{2,2} X_{2,2_{n_2}}}{K_{3,n_2} + S_{2,2}} && \text{lactate utilization} \\
& + Y_{4,4} \sum_{n_3}^{N_3} \frac{\kappa_{4,n_3} S_{2,3} X_{2,3_{n_3}}}{K_{4,n_3} + S_{2,3}} && \text{acetogenesis} \\
& + \left(\frac{V_l}{V_m}\right) \gamma_{1,4} S_{1,4} && \text{attachment} \\
& - \gamma_{2,4} S_{2,4} && \text{absorption}
\end{aligned}$$

Propionate ($S_{2,5}$):

$$\begin{aligned}
\partial_t S_{2,5} = \quad & Y_{5,2} \sum_{n_1}^{N_1} \frac{\kappa_{2,n_1} S_{2,1} X_{1,1_{n_1}}}{K_{2,n_1} + S_{2,1}} && \text{sugar utilization} \\
& + Y_{5,3} \sum_{n_2}^{N_2} \frac{\kappa_{3,n_2} S_{2,2} X_{2,2_{n_2}}}{K_{3,n_l} + S_{2,2}} && \text{lactate utilization} \\
& + \left(\frac{V_l}{V_m}\right) \gamma_{1,5} S_{1,5} && \text{attachment} \\
& - \gamma_{2,5} S_{2,5} && \text{absorption}
\end{aligned}$$

Butyrate ($S_{2,6}$):

$$\begin{aligned}
\partial_t S_{2,6} = \quad & Y_{6,2} \sum_{n_1}^{N_1} \frac{\kappa_{2,n_1} S_{2,1} X_{2,1_{n_1}}}{K_{2,n_1} + S_{2,1}} && \text{sugar utilization} \\
& + Y_{6,3} \sum_{n_2}^{N_2} \frac{\kappa_{3,n_2} S_{2,2} X_{2,2_{n_2}}}{K_{3,n_2} + S_{2,2}} && \text{lactate utilization} \\
& + \left(\frac{V_l}{V_m}\right) \gamma_{1,6} S_{1,6} && \text{attachment} \\
& - \gamma_{2,6} S_{2,6} && \text{absorption}
\end{aligned}$$

Methane ($S_{2,7}$):

$$\partial_t S_{2,7} = \quad Y_{7,5} \sum_{n_4}^{N_4} \frac{\kappa_{5,n_2} S_{2,3} X_{2,4_{n_4}}}{K_{5,n_4} + S_{2,3}} I_{pH}(x) \qquad \text{methanogenesis}$$

$$+ \left(\frac{V_l}{V_m}\right) \gamma_{1,7} S_{1,7} \qquad \text{attachment}$$

$$- \gamma_{2,7} S_{2,7} \qquad \text{absorption}$$

Carbon dioxide ($S_{2,8}$):

$$\partial_t S_{2,8} = Y_{8,2} \sum_{n_1}^{N_1} \frac{\kappa_{2,n_1} S_{2,1} X_{2,1_{n_1}}}{K_{2,n_1} + S_{2,1}} \qquad \text{sugar utilization}$$

$$+ Y_{8,3} \sum_{n_2}^{N_2} \frac{\kappa_{3,n_2} S_{2,2} X_{2,2_{n_2}}}{K_{3,n_2} + S_{2,2}} \qquad \text{lactate utilization}$$

$$+ Y_{8,4} \sum_{n_3}^{N_3} \frac{\kappa_{4,n_3} S_{2,3} X_{2,3_{n_3}}}{K_{4,n_3} + S_{2,3}} \qquad \text{acetogenesis}$$

$$+ Y_{8,5} \sum_{n_4}^{N_4} \frac{\kappa_{5,n_4} S_{2,3} X_{2,4_{n_4}}}{K_{5,n_4} + S_{2,3}} I_{pH}(x) \qquad \text{methanogenesis}$$

$$+ \left(\frac{V_l}{V_m}\right) \gamma_{1,8} S_{1,8} \qquad \text{attachment}$$

$$- \gamma_{2,8} S_{2,8} \qquad \text{absorption}$$

Water ($S_{2,9}$):

$$\begin{aligned}
\partial_t S_{2,9} = \quad & Y_{9,2} \sum_{n_1}^{N_1} \frac{\kappa_{2,n_1} S_{2,1} X_{2,1_{n_1}}}{K_{2,n_1} + S_{2,1}} && \text{sugar utilization} \\
& + Y_{9,3} \sum_{n_2}^{N_2} \frac{\kappa_{3,n_2} S_{2,2} X_{2,2_{n_2}}}{K_{3,n_2} + S_{2,2}} && \text{lactate utilization} \\
& + Y_{9,4} \sum_{n_3}^{N_3} \frac{\kappa_{4,n_3} S_{2,3} X_{2,3_{n_3}}}{K_{4,n_3} + S_{2,3}} && \text{acetogenesis} \\
& + Y_{9,5} \sum_{n_4}^{N_4} \frac{\kappa_{5,n_4} S_{2,3} X_{2,4_{n_4}}}{K_{5,n_4} + S_{2,3}} I_{pH}(x) && \text{methanogenesis} \\
& + \left(\frac{V_l}{V_m} \right) \gamma_{1,9} S_{1,9} && \text{attachment} \\
& - \gamma_{2,9} S_{2,9} && \text{absorption}
\end{aligned}$$

Mucins ($I_{2,1}$):

$$\begin{aligned}
\partial_t I_{2,1} = \quad & \Lambda && \text{endogenous production} \\
& - \frac{I_{2,1} \sum_{n_1}^{N_1} \kappa_{1,n_1} Y_{1,1} X_{2,n_1}}{\left(\sum_{n_1}^{N_1} K_{1,n_1} X_{2,1_{n_1}} \right) + I_{2,1}} && \text{hydrolysis} \\
& - \gamma_{4,10} I_{2,1} && \text{sloughing}
\end{aligned}$$

Sugar Degraders ($X_{2,1,n_1}$):

$$\begin{aligned}
\forall n_1 \leq N_1 : \partial_t X_{2,1_{n_1}} = \quad & Y_{11,2} \frac{\kappa_{2,n_1} S_{2,1} X_{2,1_{n_1}}}{K_{2,n_1} + S_{2,1}} && \text{sugar utilization} \\
& + \left(\frac{V_l}{V_m} \right) \gamma_{1,11_{n_1}} X_{1,1_{n_s}} && \text{attachment} \\
& - \gamma_{4,11_{n_1}} X_{2,11_{n_1}} && \text{sloughing} \\
& - \kappa_{6,n_1} X_{2,1_{n_s}} && \text{decay}
\end{aligned}$$

Lactate Degraders ($X_{2,2,n_2}$):

$$\begin{aligned}
\forall n_2 \leq N_2 : \partial_t X_{2,2_{n_2}} + \bar{v}_l \partial_x X_{2,2_{n_2}} = \quad & Y_{12,3} \frac{\kappa_{3,n_2} S_{2,2} X_{2,2_{n_2}}}{K_{3,n_2} + S_{2,2}} && \text{lactate utilization} \\
& + \left(\frac{V_l}{V_m} \right) \gamma_{1,12_{n_2}} X_{1,2_{n_l}} && \text{attachment} \\
& - \gamma_{4,12_{n_2}} X_{2,2_{n_2}} && \text{sloughing} \\
& - \kappa_{7,n_2} X_{2,2_{n_2}} && \text{decay}
\end{aligned}$$

Hydrogen Degrading Acetogens ($X_{2,3,n_3}$):

$$\begin{aligned}
\forall n_3 \leq N_3 : \partial_t X_{2,3_{n_3}} + \bar{v}_l \partial_x X_{2,3_{n_3}} = \quad & Y_{13,4} \frac{\kappa_{4,n_3} S_{2,3} X_{2,3_{n_3}}}{K_{4,n_4} + S_{2,3}} && \text{acetogenesis} \\
& + \left(\frac{V_l}{V_m} \right) \gamma_{1,13_{n_3}} X_{1,3_{n_3}} && \text{attachment} \\
& + \gamma_{4,13_{n_3}} X_{2,3_{n_3}} && \text{sloughing} \\
& - \kappa_{8,n_3} X_{2,3_{n_3}} && \text{decay}
\end{aligned}$$

Hydrogen Degrading Methanogens ($X_{2,4,n_4}$):

$$\begin{aligned}
\forall n_4 \leq N_4 : \partial_t X_{2,4_{n_4}} + \bar{v}_l \partial_x X_{2,4_{n_4}} = \quad & Y_{14,5} \frac{\kappa_{5,n_4} S_{2,3} X_{2,4_{n_4}}}{K_{5,n_4} + S_{2,3}} && \text{methanogenesis} \\
& + \left(\frac{V_l}{V_m} \right) \gamma_{1,14_{n_4}} X_{1,4_{n_4}} && \text{attachment} \\
& - \gamma_{4,14_{n_4}} X_{2,4_{n_4}} && \text{sloughing} \\
& - \kappa_{9,n_4} X_{2,4_{n_4}} && \text{decay}
\end{aligned}$$

4 Numerical Treatment and Considerations

The described continuous model takes a structure similar to many transport models with reactions seen in Chemical Engineering problems. The combination of both non-linear reaction terms and linear exchange terms between a

fluid and stationary medium creates individual processes proceeding at different time scales, creating significant stiffness in the source terms. To integrate our stiff model, we apply a central scheme for balance laws as described in [17]. To begin, we re-write model (1) by:

$$c_t + f(c)_x = g(c) \tag{25}$$

where $f(c)$ is the flux of material (simply first-order convection in our model), and $g(c)$ is representative of stiff source terms, as to follow the standards presented in [17].

To solve numerically, we discretize Equation (25) in both space and time:

$$\Delta x = \frac{L}{N+1}, \qquad \Delta t <= \frac{\Delta x}{2\bar{v}},$$

where L is the length of the colon, and N is the number of grid points used to discretize the continuous length. The resulting discrete representation of the model (25) is presented as:

$$\begin{aligned} u^{\tau+1}_{\chi+1/2} &= \tfrac{1}{2}\left(u^{\tau}_{\chi} + u^{\tau}_{\chi+1}\right) + \tfrac{1}{8}\left(u'_{\chi} - u'_{\chi+1}\right) - \tfrac{\Delta t}{\Delta x}\left(f(u^{\tau+1/2}_{\chi+1}) - f(u^{\tau+1/2}_{\chi})\right) \\ &+ \Delta t\left(\tfrac{3}{8}g(u^{\tau+1/3}_{\chi}) + \tfrac{3}{8}g(u^{\tau+1/3}_{\chi+1}) + \tfrac{1}{4}g(u^{\tau+1}_{\chi+1/2})\right), \end{aligned} \tag{26}$$

where u^{τ}_{χ} is the approximate concentration of measured quantity [g/L] at the index τ time step and index χth location. Equation (26) solves for concentration $u^{\tau+1}_{\chi+1/2}$ at the current time index $(\tau + 1)$ on a staggered grid (center of grid nodes), requiring previous (time level τ) and intermediate (time level $\tau + 1/3$, $\tau + 1/2$) solutions at the edge of grid nodes. Model (26) is then a system of nonlinear equations that requires iterative solving.

Values at intermediate time levels, $u_\chi^{\tau+1/2}$ and $u_\chi^{\tau+1/3}$, are solved using an implicit fractional step:

$$\begin{aligned} u_\chi^{\tau+1/2} &= u_\chi^\tau + \tfrac{\Delta t}{2}\left(g(u_\chi^{\tau+1/2}) - \tfrac{f'_j}{\Delta x}\right), \\ u_\chi^{\tau+1/3} &= u_\chi^\tau + \tfrac{\Delta t}{3}\left(g(u_\chi^{\tau+1/3}) - \tfrac{f'_\chi}{\Delta x}\right), \end{aligned}$$

and the values of u'_χ and f'_χ are first order approximation of the spatial derivatives of the field and the flux, respectively. As in [17], we employ the following flux-limiter treatment:

$$\begin{aligned} u'_\chi = &\quad \mathrm{MM}(u_{\chi+1} - u_\chi - \tfrac{1}{2}D_{\chi+\frac{1}{2}}u, u_\chi - u_{\chi-1} + \tfrac{1}{2}D_{\chi-\frac{1}{2}}u), \quad \text{where} \\ D_{\chi+\frac{1}{2}}u = &\quad \mathrm{MM}(u_{\chi+2} - 2u_{\chi+1} + u_\chi, u_{\chi+1} - 2u_\chi + u_{\chi-1}), \quad \text{and} \\ \mathrm{MM}(x,y) = &\quad \begin{cases} \mathrm{sgn}(x)\cdot\min(|x|,|y|) & \text{if } \mathrm{sgn}(x) = \mathrm{sgn}(y), \\ 0 & \text{otherwise.} \end{cases} \end{aligned}$$

to approximate spatial derivatives. In summary, the approximate solution at the current time step requires the evaluations of 5 non-linear problems using the previous solution at 6 discrete edges (3 on either side).

4.1 Boundary Conditions

To complete the model, boundary conditions must be specified at the upstream end of the lumen for all dependent variables. These boundary values are analogous to the bolus composition and frequency entering the large intestine.

Because we do not explicitly model the pre-colon processes, we make use of a *black-box* representation of the upper-GI tract, modeling the transport

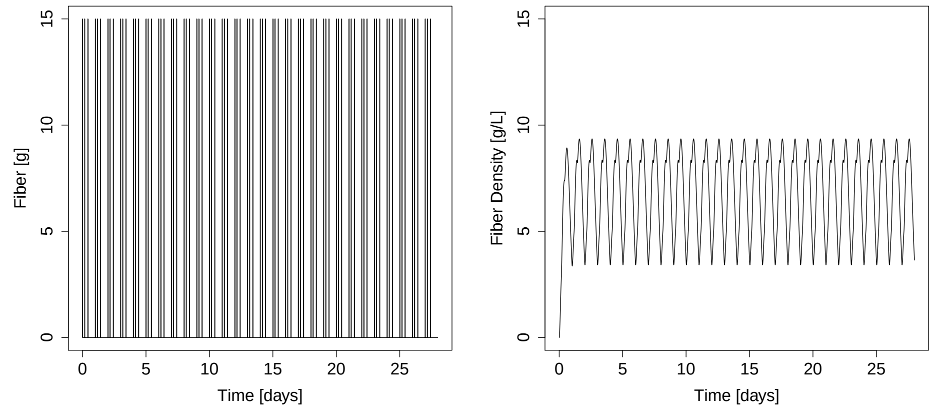

(a) Dietary Input

(b) Colon Boundary Condition

Figure 2: Effect of black-box treatment on periodic impulsed diet (3 x 15g per day, at hour 0, 4, 10 every 24 hours).

of dietary input from mouth to colon as a sequence of dilution units. This process effectively buffers sharp input conditions, which is appropriate when considering the pathway of dietary inputs traveling through the GI-tract to the colon. A sequence of dilution units is modeled as:

$$\dot{u}_1 = D(u_o - u_1) \quad \text{for first unit} \tag{27}$$

$$\dot{u}_k = D(u_{k-1} - u_i) \quad \text{for sequential units} \tag{28}$$

where D is the dilution rate found using the system flow rate and an approximate volume of pre-colon organs, and u_i is the density of material [g/L] in vessel k, with the density from the final dilution reservoir being the input to the colon model. We define the initial density into the first dilution unit u_o

as a periodic piece-wise impulse function, representative of a feeding pattern. Figure 2 demonstrates the effect of dilution treatment on an impulsive diet regiment.

4.2 Numerical Implementation

The developed mathematical model of variable problem-size and functionally defined sub-processes presents significant organizational challenges during numerical simulation. Additionally, simulation of large models will invariably create large data-sets, both with analytical and visualization challenges. The compuGUT software project stems from these design challenges, providing interested users a preliminary model implementation for review and experimentation [18](Chapter 4). Source codes, user-friendly operation and visualization scripts, additional files and resources, as well as precompiled 32 and 64bit Linux binaries are available under GNUv3 licensing at compugut.sourceforge.net.

4.3 Numerical Verification

Mass Conservation: To confirm mass conservation of the digestion submodel, numerical simulations of the model were executed. These simulations were conducted under *batch* operation assumptions (no input or output of mass), and natural decay/death of biomass is not considered. As such, the total mass of material initializing the system must equal the total mass of material at steady state. The results of this simulation scenario are presented

Table 11: Verification of mass conservation

Material	Initial Mass [g]	Final Mass [g]	Difference [g]
glucose	0	0	0
lactate	0	0	0
hydrogen	0	0	0
acetate	0	6.826067	6.826067
propionate	0	8.221962	8.221962
butyrate	0	2.666459	2.666459
methane	0	0.061338	0.061338
carbon dioxide	0	3.946864	3.946864
water	0	11.168698	11.168698
fiber	50	0	-50
SD Biomass	10	27.120000	17.120000
LD Biomass	8	9.712153	1.712153
HDA Biomass	2	2.871074	0.871074
HDM Biomass	0.5	0.935495	0.435495
Total	70.5	73.53011	**3.03011**

in Table 11.

The difference between final and initial mass is 3.03 grams. The ammonia necessary for this set of reactions to proceed given the initial fiber mass is 3.201 grams of ammonia. Therefore, 0.171 grams, or 0.2%, of unidentified material is lost during calculations. This mass lost in the system can be attributed to computational precision (rounding and truncation errors).

Spatial Discretization Errors: To verify the convergence and efficiency of the numerical implementation, we perform a grid refinement study. The

grid level, or number of discrete representations of the colon length, is given by:

$$N = 50 \times 2^g + 1, \qquad g \in [0, 1, ...5], \tag{29}$$

where g is the grid index, used to systematically generate comparable grids. Simulations were undertaken at every grid level, with continuous input conditions (versus impulsive diets discussed previously) for convenience. Additionally, simulations were run with single-species representations of each biomass group (MT-model of carbohydrate digestion) with default parameters.

Convergence is assessed by comparing the output of all dependent variables at the colon output at a specific time in the simulation (≈ 6.35 days). For ease-of-presentation, we include only the concentration of sugar utilizing biomass in Table 12. Additionally, convergence order is assessed by calculating the rate of error reduction, θ, between solutions of sequential grid resolutions as follows:

$$\theta = \frac{\left|\left|1 - \frac{f_e(X_{g+1})}{f_e(X_\infty)}\right|\right|_2}{\left|\left|1 - \frac{f_e(X_g)}{f_e(X_\infty)}\right|\right|_2}, \qquad g \in [0, 1, .., 4], \tag{30}$$

where X_g is an array of concentrations of all dependent variables at all locations for the specified grid index (g) and at the specified time (≈ 6.35 days), X_∞ is an array of concentrations of all dependent variables at the highest grid level (6), and $f_e(X)$ is an extrapolation function, taking the solutions of X at the 51 locations of the coarse most discretization scheme. The result of this convergence-order assessment is highlighted in Table 12.

Table 12: Summary of simulation results for changing grid index, g, giving total number of grid points, N. Sugar Degrading Biomass Density (SDBD) converges towards approximately 27.75 g/L at colon output, with first-order convergence (using rate of error reduction).

g	N	SDBD [g/L]	Relative Error
0	51	13.65207	0.005
1	101	13.61938	0.002
2	201	13.60245	0.001
3	401	13.59373	6e(-4)
4	801	13.58926	3e(-4)
5	1601	13.58696	9e(-5)
6	3201	13.58579	

In addition to the refinement study, evaluation of the implementation with test scenarios were assessed for accuracy and consistency through repeated simulations [18](Chapter 4).

5 Concluding Remarks

The mathematical model as constructed is a highly simplified representation of physiological mechanisms and system interplay in the colon, but uses assumptions regarding continuous flow, component exchange, and mucus representation that are comparable to *in vitro* systems currently employed in gut microflora experimentation [19, 20].

Additionally, the modeling framework is flexible and extensible, thus can be adapted to model a variety of input and initial conditions, and further

refined as more complete knowledge about physiological sub-processes is acquired.

References

[1] Tortora GJ, Derrickson BH. Principles of anatomy and physiology. John Wiley & Sons; 2008.

[2] Muñoz-Tamayo R, Laroche B, Walter É, Doré J, Leclerc M. Mathematical modelling of carbohydrate degradation by human colonic microbiota. Journal of theoretical biology. 2010;266(1):189–201.

[3] Hester RL, Iliescu R, Summers R, Coleman TG. Systems biology and integrative physiological modelling. The Journal of physiology. 2011;589(5):1053–1060.

[4] Helander HF, Fändriks L. Surface area of the digestive tract-revisited. Scandinavian journal of gastroenterology. 2014;49(6):681–689.

[5] Sadahiro S, Ohmura T, Yamada Y, Saito T, Taki Y. Analysis of length and surface area of each segment of the large intestine according to age, sex and physique. Surgical and Radiologic Anatomy. 1992;14(3):251–257.

[6] Cummings J, Macfarlane G. The control and consequences of bacterial fermentation in the human colon. Journal of Applied Bacteriology. 1991;70(6):443–459.

[7] Batstone DJ. Mathematical modelling of anaerobic reactors treating domestic wastewater: Rational criteria for model use. Reviews in Environmental Science and Biotechnology. 2006;5(1):57–71.

[8] Batstone DJ, Keller J, Angelidaki I, Kalyuzhnyi S, Pavlostathis S, Rozzi A, et al. The IWA Anaerobic Digestion Model No 1(ADM 1). Water Science & Technology. 2002;45(10):65–73.

[9] Gusakov A, Sinitsyn A, Klyosov A. Kinetics of the enzymatic hydrolysis of cellulose: 1. A mathematical model for a batch reactor process. Enzyme and microbial technology. 1985;7(7):346–352.

[10] Vavilin V, Rytov S, Lokshina LY. A description of hydrolysis kinetics in anaerobic degradation of particulate organic matter. Bioresource technology. 1996;56(2):229–237.

[11] Eastman JA, Ferguson JF. Solubilization of particulate organic carbon during the acid phase of anaerobic digestion. Journal (Water Pollution Control Federation). 1981;p. 352–366.

[12] Vavilin V, Fernandez B, Palatsi J, Flotats X. Hydrolysis kinetics in anaerobic degradation of particulate organic material: An overview. Waste management. 2008;28(6):939–951.

[13] Stewart EJ, Madden R, Paul G, Taddei F. Aging and death in an organism that reproduces by morphologically symmetric division. PLoS biology. 2005;3(2):e45.

[14] Ramirez I, Volcke EI, Rajinikanth R, Steyer JP. Modeling microbial diversity in anaerobic digestion through an extended ADM1 model. Water research. 2009;43(11):2787–2800.

[15] Molly K, Woestyne MV, Verstraete W. Development of a 5-step multichamber reactor as a simulation of the human intestinal microbial ecosystem. Applied Microbiology and Biotechnology. 1993;39(2):254–258.

[16] Press WH, Teukolsky SA, Vetterling WT, Flannery BP. Numerical recipes in C: the art of scientific computing, 1992. Cité en. 1992;p. 92.

[17] Liotta SF, Romano V, Russo G. Central schemes for balance laws of relaxation type. SIAM Journal on Numerical Analysis. 2000;38(4):1337–1356.

[18] Moorthy AS, Brooks SP, Kalmokoff M, Eberl HJ. compuGUT: An in silico tool for studying flora composition in the human large intestine; 2015. Http://compugut.sourceforge.net/preprint.

[19] Macfarlane GT, Macfarlane S. Models for intestinal fermentation: association between food components, delivery systems, bioavailability and functional interactions in the gut. Current opinion in biotechnology. 2007;18(2):156–162.

[20] Payne AN, Zihler A, Chassard C, Lacroix C. Advances and perspectives in in vitro human gut fermentation modeling. Trends in biotechnology. 2012;30(1):17–25.
